# Identification of threshold concepts in the undergraduate orthodontics curriculum: a modified Delphi study

**DOI:** 10.1186/s12909-025-08516-6

**Published:** 2026-01-09

**Authors:** Aisha Sher, Rahila Yasmeen, Ulfat Bashir

**Affiliations:** 1 Medical Education Department, Margalla College of Dentistry, Rawalpindi, Pakistan; 2 Riphah Academy of Research & Education (RARE), Riphah International university, Islamabad, Pakistan; 3 Riphah International University, Islamabad, Pakistan; 4 Islamic International Dental College, Islamabad, Pakistan

**Keywords:** Threshold concepts, Undergraduate orthodontic curriculum, Teaching strategies, Assessment strategies, Transformative, Troublesome

## Abstract

**Background:**

Orthodontics is acknowledged as an integral clinical subspecialty in undergraduate dental programs. Mastering such an extensive and multifaceted theoretical knowledge and its implementation into practice is challenging. The student experiences tension, difficulty and frequently lacks preparedness and confidence when performing different orthodontic procedures. Threshold concepts (TCs) can offer a potential solution to this problem. The transforming nature of threshold concepts can significantly influence student learning and curriculum development. A number of learning theories resonate with the concept of TCs. The study aimed to explore TCs and associated teaching strategies (TS) and assessment strategies (AS) in the undergraduate orthodontic curriculum through expert consensus, offering reforms in dental education.

**Methodology:**

Phase 1 of the study comprised a thorough literature review followed by a preliminary online session with experts to outline the curriculum and assess their understanding of TCs. Two orientation sessions for participants and three FGDs were conducted in two different institutes of twin cities in Pakistan. Qualitative analysis of data was done to formulate questionnaire for Modified Delphi Round 1. In phase 2, the Modified Delphi technique was used to obtain consensus on 39 proposed TCs, their 26 teaching and 26 assessment strategies under nine domains of orthodontics. 41 experts with postgraduate degrees in orthodontics and a minimum of 2 years of teaching experience in the field participated in round 1, 38 in round 2 and 35 in round 3. Prior termination criteria were set with 3 Delphi rounds, ≥80% agreement and stability. Items with ≥80% agreement and stability of responses between two consecutive rounds reached consensus.

**Results:**

34 out of 39 TCs reached consensus. 5 TS and 4 AS were identified for (domains I and II) patient assessment. Similarly, 2 TS and 4 AS for growth and development (domain III), 6 TS and 5 AS for diagnosis (domains IV & V), 6 TS and 5 AS for treatment of orthodontic patients (domains VI, VII & VIII), 3 TS and 1 AS for recent advances (domain IX) reached consensus.

**Conclusion:**

The identified TCs have highlighted specific areas in orthodontics that may pose challenges for students and prevent learning. Due consideration to these TCs and appropriate TS and AS can transform students from novices to experts. The study offered a comprehensive TC framework that can guide undergraduate orthodontic curriculum and practices.

**Supplementary Information:**

The online version contains supplementary material available at 10.1186/s12909-025-08516-6.

## Introduction

Orthodontics is the subspeciality of dentistry that deals with the growth and development of craniofacial region, diagnosis, treatment, prevention, and correction of misaligned teeth, jaws, and bite patterns. The correction of face growth, or dentofacial orthopaedics, may also be included. The American Association of Orthodontics estimates that 50% of population in affluent nations have significant malocclusions, necessitating orthodontic treatment. Worldwide undergraduate dentistry programs aim to provide students with the basic understanding and proficiency in orthodontics, a recognized dental specialty, so they can properly treat patients. On the other hand, the breadth of orthodontics education differs both nationally and internationally among undergraduate institutions. This results in disparities in curricular content as well as teaching and evaluation strategies.

Consequently, there could be discrepancies in the way dental graduates demonstrate orthodontic skills in general practice, including diagnosis, patient management, and referral. The diagnosis, timely referral, and efficient care of orthodontic patients are greatly aided by GDPs. As per a survey, in the USA 72% of general dentists provide some form of orthodontic treatment [1]. The Association for Dental Education in Europe (ADEE) urges dental graduates from Europe, upon graduating, to exhibit the ability to diagnose orthodontic treatment needs, use contemporary treatment methods, handle all types of orthodontic emergencies, and make referrals as needed [2, 3].

The American Dental Association’s (ADA) Commission on Dental Accreditation (CODA) mandates that predoctoral graduates give patients comprehensive care appropriate to their level of expertise in nearly every area of general dentistry practice related to space management protocols and standards of malocclusion [1].

Threshold concepts (TCs), first proposed by Meyer and Land in 2003, are the concepts in a discipline, referred as portals/learning spaces to be crossed, that results a shift in learner’s perspective once they understand and master these difficult concepts [4]. Learning threshold concepts requires the learner to battle through a transitory state known as liminal space, as they attempt to acquire new understandings. The word “limen” is Latin for threshold. Being in a transitional state is like being just on the cusp of something new, but not quite there [5]. Within the liminal phase, learners integrate new knowledge by reconfiguring conceptual frameworks and discarding earlier understandings. This process triggers both ontological and epistemic shifts, which are reconstitutive elements of threshold concept, collectively enabling new understanding [6].

Most of the literature is based upon five main characteristics of TCs. They can be *transformative* (once understood they results in a conceptual shift), *integrated* (revealing interrelatedness of something previously hidden or unnoticed), *bonded* (discipline specific), irreversible (difficult to unlearn once grasped) and *troublesome* (learner may find them difficult to understand or they challenge their previous beliefs) besides *discursive* (enable learner to appreciate and use appropriate language) and *reconstitutive* (the integration/reconfiguration of knowledge and subsequent ontological/epistemic transition) [4, 6] Annexure I, Additional file 1(Operational definitions). These characteristics distinguish TCs from key/core concepts of a discipline [7]. These can inform how curricular content can be mapped (identifying transformative, troublesome and integrating aspects), contextualized (bonded as well as integrated and irreversible aspects), presented (teaching strategies and scaffolding) and assessed [8].

These can be defined as the process of developing a professional identity, knowledge, and skills. Until now, this perspective has not been considered in the literature on dentistry [5, 9]. Furthermore, there are significant gaps in the information currently available regarding the TCF’s potential for use in education and its assessment, both of which require additional study [10].

### Problem statement

Curricula for orthodontic undergraduate education that encompass a variety of topics from the preclinical, clinical, and theoretical domains are rarely regarded as ideal. It is often packed with vague or precise educational objectives, and students often don’t fully grasp a subject until they have acquired specific information and experience. The standard curriculum offers little opportunity for changing the ways in which knowledge is distributed because it is based on conventional learning methodologies like apprenticeship models and didactic learning. The training needs in relation to the difficulties faced by undergraduate orthodontic students in understanding key concepts, duration of program, number of skills transferred/inadequate exposure and lack of preparedness and confidence while performing various orthodontic procedures, have received very little attention [11, 12].

Another fundamental problem in education has been identified as how to develop the relationship between the two complementary knowledge structures, which is hampered in the clinical setting by the so-called “theory-practice gap.” There are two types of knowledge structures in clinical dentistry, i.e., (experiential/linear and conceptual/hierarchical. To achieve expertise, it is crucial to link complementary knowledge structures. For example, to successfully register jaw relation for complete denture one must be able to link underlying theory with practical sequence [13]. The distance between theory and practice may be overcome by threshold concepts, and dental education will be improved. There is also limited literature with a focus on technical cognitive tasks in medical education [14].Despite the fact that numerous dental experts have underlined the significance of threshold concepts and their implications in dental education, these concepts have yet to be recognized.

### Rationale of study

The study will examine threshold concepts that are crucial for undergraduates in the orthodontics dental curriculum. Threshold concepts have a transformative capacity that is important for curriculum development and student learning. Students may be able to progress through a program more quickly and effectively if the curriculum is designed/modified around the threshold concepts of the discipline. It can also help students navigate the educational landscape [15].Additionally, this will assist students in effectively bridging the theory-practice divide.

If these ideas are grasped, we may create a curriculum that includes “thresholds to be crossed” rather than merely “content to be covered” [16].

As TCs can inform teaching and learning practices, they have been referred to as curriculum jewels in the literature. Therefore, it is essential to identify and address TCs in all disciplines. Evidence exists for the regular application of TCs in the humanities and STEM (science, technology, engineering, and mathematics) domains. TCs have been recognized in a variety of health professions educational contexts, involving medical, nursing and physiotherapy students as well as at the postgraduate level among physicians, nurses, pharmacists, and occupational therapists. TCs may be very beneficial in helping students better understand the relationships between the formal and hidden curriculum, develop new ways of thinking and practicing, and gain new perspectives on the environment and types of learning involved. A threshold concept framework can also be employed as a strategy to promote communication and discussion among educators, teachers and learners, which can be alternatively termed “transactional curriculum inquiry. Educators can decide precisely what is essential for students to understand their subject by focusing on threshold concepts [5].

**Fig. 1:**
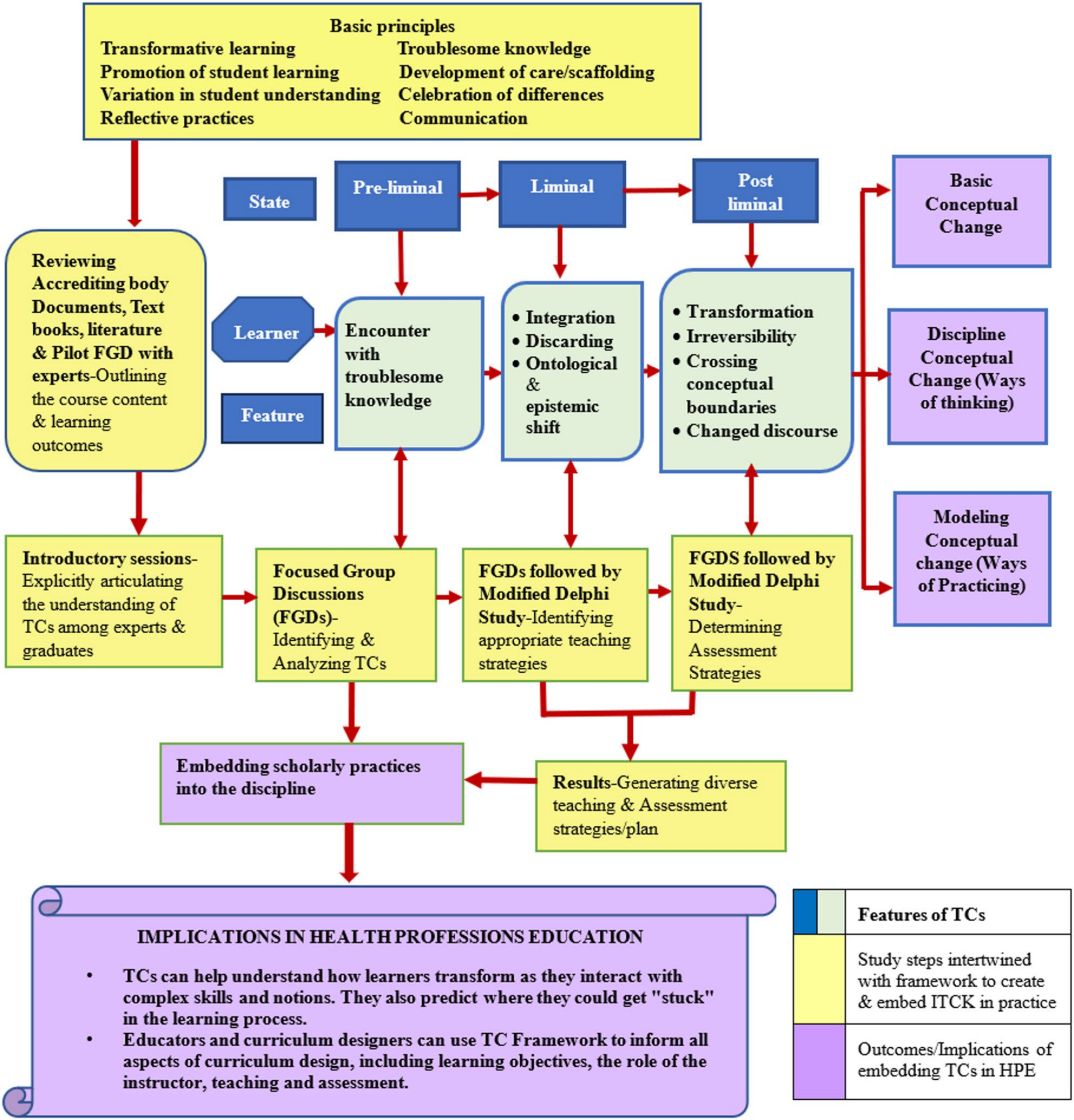
Conceptual framework informed by Threshold Concepts and transformational learning (Meyer et al., Brill Publishers) & The Framework to create & embed Integrated Threshold Concept Knowledge (ITCK) (Timmermans & Meyer, International Journal for Academic Development) [6, 17]

The aim is to improve learning experiences of students through appropriate teaching strategies and assess their learning via methods that documents observable signs of conceptual improvement in students’ understanding, ways of behaving and practicing. Fig.1 represents the framework for this study.

The objectives of the study are *to identify threshold concepts in orthodontics’ undergraduate dental curriculum* and *to determine the teaching and assessment strategies of identified threshold concepts in the orthodontic undergraduate dental curriculum.*

**Fig. 2:**
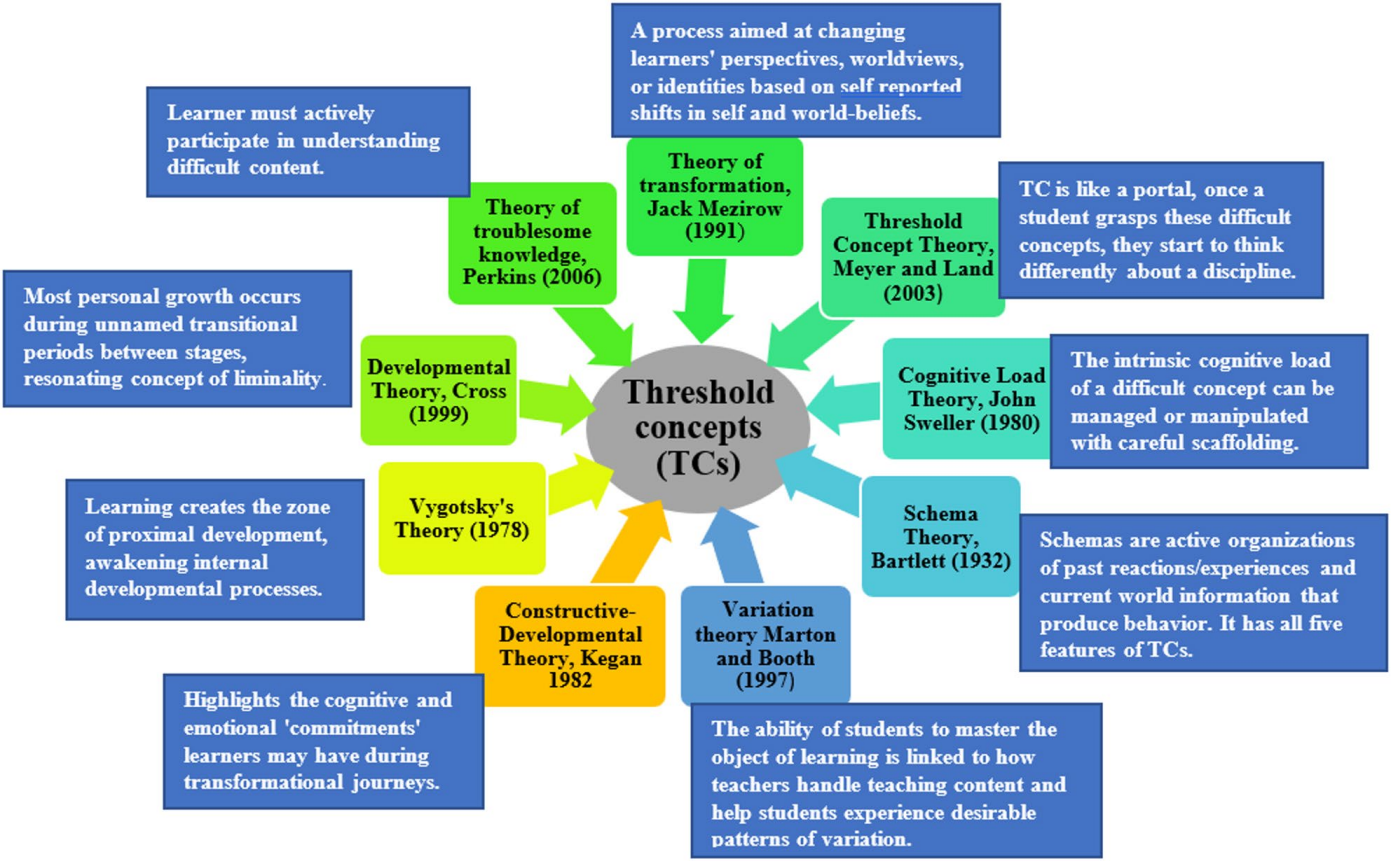
Connection of Threshold concept with various learning theories-based upon existing literature [6, 19–23]

The threshold concept theory will serve as the fundamental conceptual framework underpinning this study. This theory presents the notion of liminality as a conceptual space that is provoked by the learner’s interaction with a TC, as they move through the space, they start to transform [18]. A number of learning theories resonate with the notion of threshold concept as indicated in Fig. 2.

All these theories, which operationalize TCs through certain frameworks or lenses to improve usage, are valid. Researchers found them applicable to dentistry [24].

## Methodology

A thorough literature review was carried out regarding work done on TCs in Dentistry with special consideration to orthodontics, using three data bases namely PubMed, Psych INFO and ERIC. Google Scholar was consulted for grey literature.

### Study design and settings

In the absence of previous orthodontic specific TC definitions, we extended the conceptual framework and characteristics of TCs originally developed by Meyer and Land, in higher education and later applied in medicine and other health sciences, to the context of orthodontic undergraduate curriculum and learning outcomes. These were then validated by expert deliberation in Delphi process, consistent with Barradell’s recommendations for domain specific TC identification [8].

**Fig. 3:**
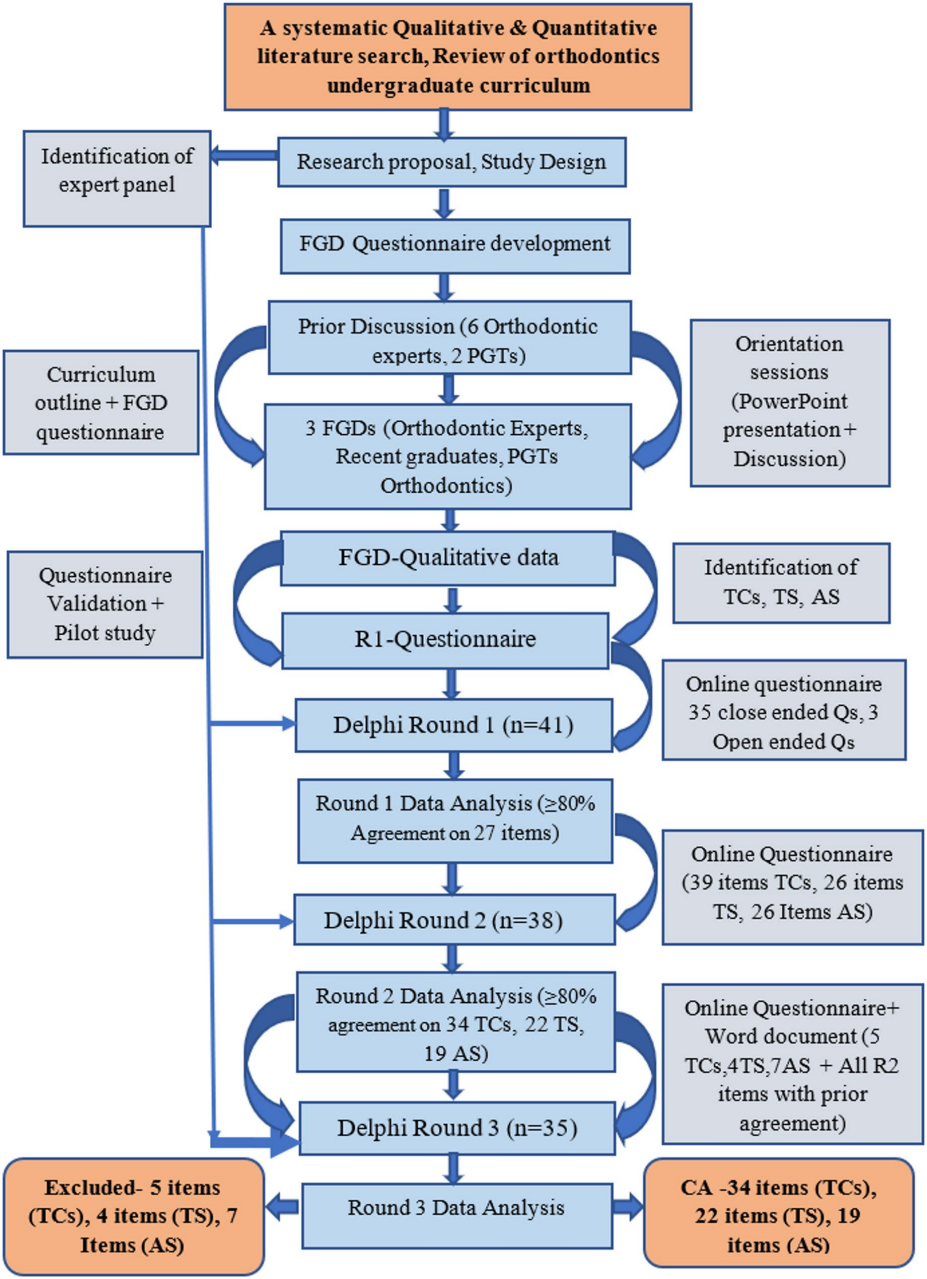
Overview of the study

A modified Delphi (mixed-method) technique was employed with a preliminary Phase that involved stages to outline curriculum and conduct face to face Focus Group Discussions (FGDs) in 2 different Institutes of twin cities of Pakistan. Figure 3.

Duration of study was 8 months from February 2023 to September 2023. Ethical approval was granted by the Islamic International Medical College Ethical Review Committee under reference number Riphah/IIMC/IRC/23/3023. Confidentiality of participant was ensured throughout the research process.

## Phase I

### Materials and methods

#### Curriculum outline

As undergraduate orthodontic curriculum **v**aries from one institute to another [1], it was outlined with the help of guidelines provided by PMDC and different universities as well as consulting subject experts, course and reference books [25, 26]. The curriculum was split into three main areas: growth and development, patient assessment and diagnosis, and treatment of orthodontic patient, for ease of participants and cover all aspects.

A questionnaire for FGDs was initially developed by reviewing literature. A preliminary online session involving subject matter experts and medical educationists from different institutes was conducted to assess experts’ knowledge of TCs and modify the undergraduate orthodontic curriculum outlined earlier from literature. The discussion also helped to modify questionnaire to only few questions according to the demand of study and determine probes for further FGDs [27]. Two subject matter experts and two medical educators validated the questionnaire. Questionnaire, Annexure I-Supplementary file 1.

Orientation sessions were conducted a week before FGDs to familiarize participants from each institute with TC theory and orthodontic curriculum. Reading material was provided to understand the concepts. This also gave them time to reflect on their experiences.

#### Focus group discussions (FGDs)

Informed consent was obtained from all the participants before the commencement of each FGD, Annexure I & II, Related file 1. 1^ST^ and 2^nd^ FGD conducted in the same institute, comprised of 9 participants (3 Orthodontic experts and 4 PGTs) participated in 1 ^st^ and 2^nd^ FGDs. 1 ^st^ FGD was intended to identify Threshold concepts while 2^nd^ FGD aimed at discussing teaching and assessment strategies of the identified TCs.

3^rd^ FGD conducted in a different institute, had 6 participants (2 fresh graduates, 4 PGTs). Heterogenous group of participants was engaged to generate a transactional curriculum inquiry [7, 28]. Undergraduate students were not included in FGDs as they had not yet been exposed to the complete undergraduate curriculum and had limited clinical exposure.

FGDs promoted discourse, discussion, and communication—all of which are beneficial for gathering diverse perspectives and discussing TCs, a relatively ethereal and complex domain [29]. Participants were requested to reflect on their undergraduate experiences and point out the concepts from the given orthodontic curriculum that fulfill two or more characteristics of a TC, one of these should either be transformative or troublesome. The FGDs were limited to identification of TCs their teaching and assessment strategies for this study owing to length of undergraduate curriculum. Discussion was recorded on two separate devices. Curriculum outline was displayed for the ease of participants so that they may not skip any part. The participants were verbally probed during the process.

### Qualitative data analysis

Language limitations necessitated manual transcription of the data. The naturalized transcription (intelligent verbatim) method was used [30]. Thematic analysis was done by thoroughly studying separate transcripts for emergent themes. Required areas from transcripts were compared and refined depending upon frequency of appearance [31, 32].

#### FGD results

**Fig. 4:**
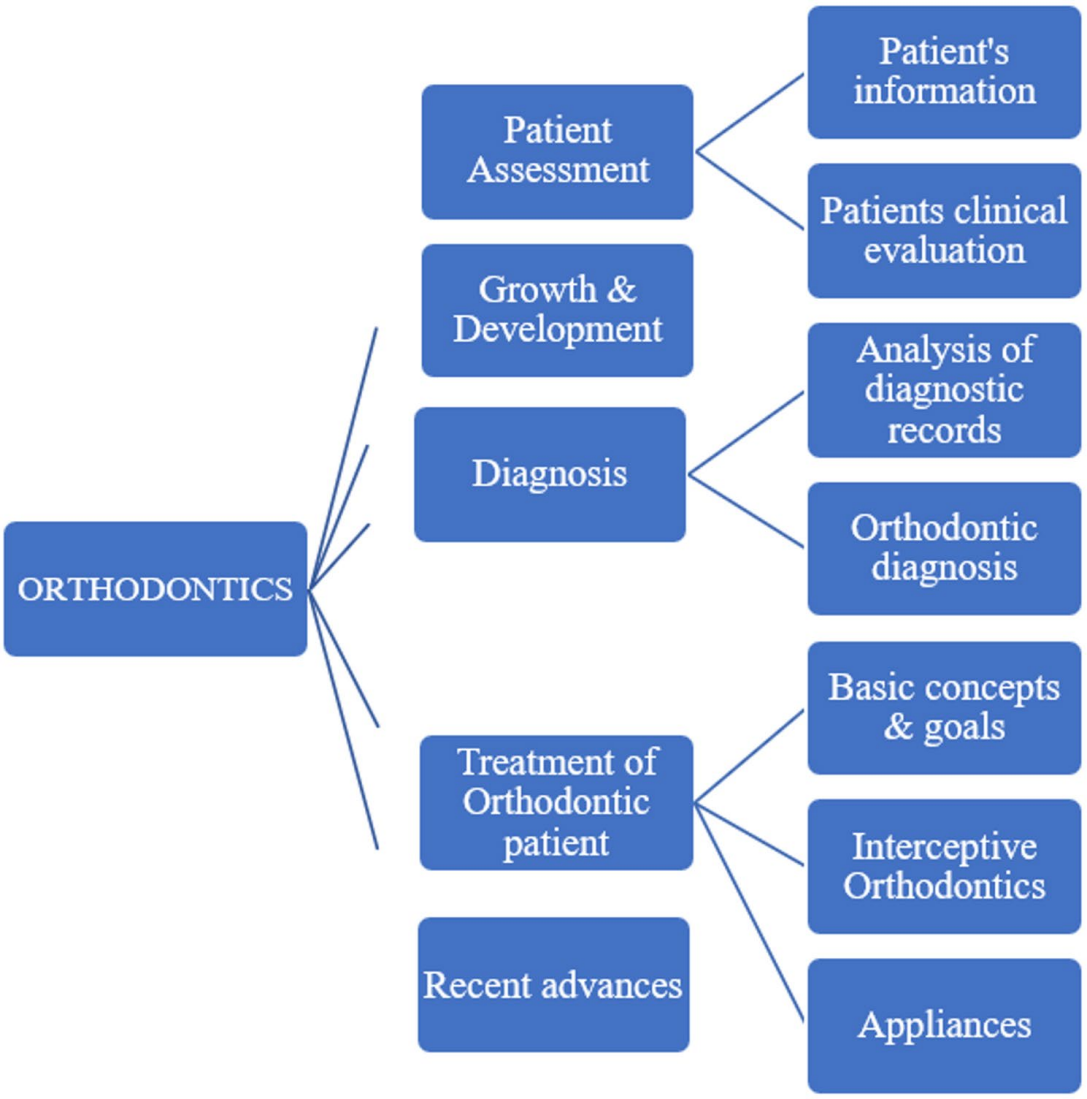
Breakdown of orthodontic curricula into domains/themes, derived from FGDs

Summary of 3 FGDs was arranged in the form of a table with derived themes/domains and proposed TCs, TS and AS. Annexure-II, Supplementary file 2. The content validity was established by refining and organizing the proposed items under the five areas and total nine domains after consultation with subject matter experts and educationists, Fig. 4; Table 1.

**Table 1:** Proposed TCs for round 1 Modified Delphi questionnaire

Sr#	Domains/Themes	Proposed Threshold Concepts
I	**Patient’s information**	1.Taking history of Orthodontic patient
II	**Patient’s Clinical Evaluation**	2.Soft tissue paradigm 3. Facial profile analysis
III	**Growth and development**	4.Growth pattern of Mandible and Maxilla 5.Significance of mandibular rotations 6.Various growth indicators determining the peak skeletal growth/Growth Assessment parameters
IV	**Analysis of diagnostic records**	7.Arch dimensions (Arch length, width, depth & Curve of Wilson, Spee, Monson) 8.Eyeballing method of calculating ALD 9.Bolton’s analysis 10.OPG analysis/Dental age analysis of OPG 11.Analysis of CVM stages on lateral Cephalogram 12.Limitations of ANB angle 13.Composite Mixed dentition analysis
V	**Diagnosis**	14.Making a problem list 15.Diagnosis of growth and developmental disorders 16.Diagnosis of impacted maxillary canines.
VI	**Treatment of orthodontic patient- Basic concepts and goals**	17.Treatment planning of various Malocclusions 18.Significance of molar (healthy/mutilated) relationship 19.Extraction patterns (molar/premolar/Incisor) in treatment planning 20. Balancing and compensating extractions of 6s. 21.Periodontal considerations for orthodontic treatment 22.Basics of biomechanics 23.Basics of Anchorage control in orthodontics 24.Retention and Relapse 25.Clinical Implications of developmental disorders in orthodontics 26.Knowing when and whom to refer 27.Iatrogenic effects of orthodontic treatment
VII	**Interceptive orthodontics**	28.Management of 1 ^st^ molars with poor prognosis 29.Interception of incisor Trauma 30.Mixed dentition space management protocols (space maintenance/regaining, supervision/serial extraction)
VIII	**Appliances**	31.Indications of various appliances (Removable/functional/fixed) 32.Bracket placement 33.Prescriptions of brackets
IX	**Recent Advances**	34.Concept of clear aligners 35.Basic concept of CBCT

### Phase II

A modified Delphi (mixed-method) technique was used to reach consensus and validate items put out in earlier FGDs.

### Materials and methods

The modified Delphi method is a structured, iterative approach of acquiring expert opinions and incorporating controlled feedback until a consensus is reached [33, 34]. This approach has been used to identify competencies, curriculum components and management in health care that lack empirical evidence, assessment instruments, and even threshold concepts in the medical education [35–37]. The design of Delphi is usually dictated by the research problem instead of the procedure’s requirement [38]. A thorough literature review reveals that there are no universally agreed guidelines for conducting (for standardizing technique, the number of rounds, criteria for selecting experts and the consensus) and reporting Delphi till date [33, 39]. It is a flexible technique that can be modified according to the design and purpose of research while maintaining research rigor, fundamental principles, and precision [38, 40]. The study adhered to CREDES guidelines [41, 42] **.**

### Questionnaire

The questionnaire for Delphi Round 1 was developed from items proposed during FGDs, expert recommendations and literature guidelines [43]. It was sent to three orthodontic experts and medical educationists. Items were added, deleted and modified and format was changed based on experts’ recommendations to make questionnaire more precise and comprehensible.

First section of questionnaire included information about the TC, instructions, informed consent, Participant’s demographic data and contact information. It consisted of 35 structured questions (proposed TCs) within nine orthodontic domains, comments/suggestion section and three open ended questions to suggest TS, AS and missing TC. A three-point Likert scale (agree, disagree, or indecisive) was employed since it was deemed adequate, practical, and easy to use [44].

Meyer and land suggested five main characteristics of TCs beside others but they did not specify a number or combination required by a concept to be a TC, though variation exists in literature. A prior criterion established in consultations with experts was mentioned in the questionnaire, that in order to qualify as TC an item must possess at least two of the characteristics of TC, one of these should be transformative or troublesome as these two were deemed prominent during literature review [4]. Annexure I, Suplementary file 2 (Operational definitions).

### Pilot study

Initially, a pilot study with 12 participants was carried out (six orthodontic experts responded to initial questionnaire and six PGTs responded to formatted questionnaire). Most participants found list of concepts comprehensive, relevant and explicit.

### Consensus in Delphi

In Delphi research, consensus criteria have never been formally established. Some scholars believe that the definition of consensus is one of the most delicate methodological entities and should be addressed before the process starts.

When intended agreement on a particular matter is obtained, consensus takes place. Once agreement is established, a study can be concluded. A widely accepted definition of agreement is commonly defined when at least 70% of respondents (a predetermined cut-off) either agree or disagree with a response [42]. Items were included or excluded depending upon percentage agreement or disagreement, and the items that don’t fit this criterion should be passed to successive rounds for re-consideration. The criterion of agreement varies widely, ranging from 51 to 80% [33, 45]. The majority of studies reported two or three rounds as a prior decided criteria to establish consensus in modified Delphi studies [39, 46]. 

### Closing criteria

Following closing criteria was established in advance.

Round 1: ≥ 80% expert agreement is required to reach consensus.

Round 2: ≥ 80% expert agreement + Stability between 1^st^ & 2^nd^ round.

Round 3: ≥ 80% panel agreement + Stability between 2^nd^ & 3^rd^ round.

The main decision criteria was the percentage agreement among participants. The study was restricted to three rounds in order to prevent expert attrition and respondent weariness.

### Stability

The stability of the results was established in order to improve the results’ rigor and authenticity. It is defined as “the consistency of responses between successive rounds of a study”. Stability is said to be achieved if the outcomes of the two separate Delphi rounds do not show statistically significant difference. McNamar chi square, a non-parametric test was used to check the change in experts’ responses between the two successive rounds of Delphi [47]**.** A *p*-value greater than 0.05 indicates stability in responses [48, 49].

### Study population and sampling

The intended population consisted of orthodontic experts holding a postgraduate degree (FCPS, MDS, M-Orth, MCPS, etc.) and a minimum of two years of undergraduate teaching experience. The purposive sampling technique also known as judgmental/expert sampling was used [33, 43]. LinkedIn was used to find international experts. The experts from private, semi-private, and public institutes all over Pakistan were scrutinized through PAO database.

The sample size might vary from 07 to 30, and response rates within disciplines can change based on the research interests of the members [46, 50]. An optimal double-digit number for a homogeneous Delphi is usually considered to be between 30 and 50 in the final rounds [39].

### Response rate for modified Delphi study

Invitations were sent to 55 subject experts via WhatsApp and emails along with the information sheet, Questionnaire and reading material. 41 experts participated in 1 ^st^ round, 38 in 2^nd^ and 35 in the 3rd round with the response rate of 85.41%, 92.6 and 92.1% respectively.

### Delphi round 1

#### Data collection

The form consisted of 35 structured questions regarding proposed TCs and 3 open ended questions to account for TS, AS and missing TCs as explained in questionnaire section above, Annexure II-Supplementary file 1. In addition, a PowerPoint presentation, voice note and reading material was sent to participants to comprehend TCs.

#### Data analysis

SPSS (Statistical Package for Social Scientists) was used to perform a descriptive analysis. The percentages and frequency of the responses were calculated. The stability of items receiving 80% or more agreement, was determined after the subsequent round.

Open-ended questions were subjected to qualitative data analysis.

### Delphi round 2

#### Data collection

An online questionnaire consisted of 27 items with 80% or more agreement in Round 1 (to determine stability), 04 new TCs suggested by experts and 08 items that didn’t reach consensus in Round (1) Teaching and assessment strategies were also included based upon round 1 experts’ response and FGDs conducted earlier. A 3-point Likert scale was used. Estimated time to fill the form was 25–30 min. Controlled feedback in the form of individual response against group responses (participant information sheet) was also sent to participants so that they can reassess their prior responses, Annexure III- Supplementary file 1. Experts were given 2 weeks to respond.

#### Data analysis

SPSS version 26 was used to perform descriptive analysis. Stability of responses between Round 1 and Round 2 was determined using McNemar Test. P value > 0.05 indicates that there is no significant difference between the results of round 1 and 2.

### Delphi round 3

#### Data collection

The questionnaire was sent as a Word document in addition to online google form. It consisted of all the items from round 2 along with individual and group responses. The experts rephrased 2 items, Annexure IV- Supplementary file 1. Participants were given 2 weeks to re-evaluate their responses.

#### Data analysis

For quantitative-descriptive analysis and measure of stability, data was manually entered into Excel sheets and then imported into SPSS. The final list of items (TCs, TS and AS) was compiled.

## Results

Detailed results with percentage agreement and stability attached in Annexure-III & IV- Supplementary file 2.

### Round 1

27 out of 34 items reached ≥ 80% agreement.

#### Qualitative analysis

4 new TCs were proposed. 3 (Occlusion, etiology of malocclusion and biology of tooth movement) were placed under growth and development and one (Ethical practice) under treatment of orthodontic patients.

05 teaching strategies and 06 Assessment strategies were paired under domain of patient Assessment, 05 TS and 05 AS for Growth and development, 06 TS and 06 AS under Diagnosis, 06 TS and 06 AS under the treatment domain, 04 TS and 03 AS for Recent Advances) through qualitative analysis.

### Round 2

5 TCs- Eye balling method of calculating ALD, Limitation of angle ANB, Bracket Placement, Prescription of brackets, Concept of clear aligners, MCQs, SEQs, mini-CEX, Viva Voce- Domain I&II, Clinical clerkship, Flipped Classroom, SEQs-Domain III, Interactive Lectures, SEQs- Domain IV & V didn’t reach ≥ 80% agreement.

### Round 3

After round 3, 34 out of 39 TCs reached consensus with TS and AS, as given in Table 2. 

**Table 2:** Recommended TCs with their TS and AS at the conclusion of the modified Delphi study

Threshold Concepts	Teaching Strategies	Assessment Strategies
**I-Patient’s information** 1. Taking history of Orthodontic patient **II- Patient’s Clinical Evaluation** 2. Soft tissue paradigm 3. Facial profile analysis	1.Clinical clerkship/Chair side teaching 2.SGD (simulated patients, Role play, tutorial, CBL) 3. Demonstration 4. Case presentation	1. OSCE 2. TOACS
**III- Growth and development** 4. Growth pattern of Mandible and Maxilla 5. Significance of mandibular rotations 6. Various growth indicators determining the peak skeletal growth/Growth Assessment parameters 7. Occlusion 8. Biology of tooth movement 9. Etiology of Malocclusion	1.Interactive Lecture 2.SGD (Tutorials, CBL)	1.MCQs 2.SAQs 3.OSCE 4.Viva Voce
**IV- Analysis of diagnostic records** 10. Arch dimensions (Arch length, width, depth & Curve of Wilson, Spee, Monson) 11. Bolton’s analysis 12. OPG analysis/Dental age analysis of OPG 13. Analysis of CVM stages on lateral Cephalogram 14. Mixed dentition analysis **V- Diagnosis** 15. Making a problem list 16. Diagnosis of growth and developmental disorders 17. Diagnosis of impacted maxillary canines	1. SGD (Tutorials, CBL) 2. Demonstrations 3.Hands on activities (Assessment of Patient’s Orthodontic records) 4.Clinical Clerkship/Chairside teaching 5. Case presentations	1. MCQs 2. Clinical Targets 3. OSCE 4. Viva Voce 5.TOACS
**VI- Treatment of orthodontic patient- Basic concepts and goals** 18. Treatment planning of various Malocclusions 19. Significance of molar (healthy/mutilated) relationship 20. Extraction patterns (molar/premolar/Incisor) in treatment planning 21. Balancing and compensating extractions 22. Periodontal considerations for orthodontic treatment 23. Basics of biomechanics 24. Basics of Anchorage control in orthodontics 25. Basics of Retention and Relapse 26. Clinical Implications of developmental disorders in orthodontics 27. Knowing when and whom to refer 28. Ethical practice 29. Iatrogenic effects of orthodontic treatment **VII- Interceptive orthodontics** 30. Management of 1 ^st^ molars with poor prognosis 31. Interception of incisor Trauma 32. Mixed dentition space management protocols (space maintenance/regaining, supervision/serial extraction) **VIII- Appliances** 33. Indications of various appliances (Removable/functional/fixed)	1.Interactive Lecture 2.SGD (Tutorials, CBL) 3.Demonstrations 4.Hands on activities (Assessment of Patient’s Orthodontic records) 5.Clinical Clerkship/Chairside teaching 6.Case presentations	1.MCQs 2.SAQs 3.OSCE 4.Viva Voce 5.DOPS
**IX- Recent Advances** 34. Basic concept of CBCT	1.Interactive Lecture 2.SGD	1.MCQs

The final list consists of two categories of items:

1. Items that met agreement and showed stability of responses: These items are recommended to be included in curriculum and practices to improve student learning.
2. Items that exhibit stability of responses but did not reach agreement of 80% or above: These items are excluded from the list of proposed TCs, TS and AS.

Graphical representation of results is given in Fig. 5, Supplementary file 3.

### Qualitative analysis of open-ended questions

Majority of the experts agreed that the proposed list of TCs is comprehensive and agreed that the most of these teaching and assessment strategies can be employed for undergraduate students. AU “These are the strategies I would prefer”. Experts were of the opinion that; CRQ “At the undergraduate level, scientific, logical, and practical procedures shall be emphasized. Later, at the house job or internship, the fresh graduates could use their theoretical knowledge to gain advanced practical application”, “Basics have to be strong”. “Clinical clerkship/chairside teaching of the above shall be emphasized rather than theoretical assessment”. HP “Clinical interactive teaching on actual patient records to be encouraged”.

SA “As far as recent advances are concerned chairside teaching is a good idea for aligners but for CBCT if only basics are discussed there, normally at chairside we touch higher cognition. Viva Voce for aligners is fine not for CBCT”.

Among all concepts, the prescription of brackets was least recommended and pointed out by most experts as not a TC at the undergraduate level. AI “For an undergraduate student, different prescriptions of brackets may not be important”. Similarly flipped classroom and SEQs did not reach agreement. WJ “Flipped classrooms are over-rated”. SN “Seqs least recommended”.

## Discussion

The study addresses two main challenges in identification process; firstly, by engaging recent graduates, subject matter experts and educators; and secondly by achieving consensus among participants to validate the identified TCs [8]. Owing to the scarcity of literature in the field, the discussion was built on relevant studies available in other specialties and those related to the orthodontic curriculum. Threshold concepts are said to be much more as compared to core concepts that are reported as “building blocks” of a discipline but do not alter student perception [5, 51]. In an increasingly globalized, diverse, and complex higher education environment, educators continue to face challenges in their discovery of ways to promote high-quality teaching and learning. Scholarly interest in the theory of TCs has grown significantly, as educators look for better approaches to support student learning [52].

In dentistry, the identification of TCs is particularly crucial as a means of managing the overburdened dental curriculum by consolidating resources for teaching and learning in areas that most students find difficult and support more individualized student learning/transition, contrary to the “one size fits all” approach used by most universities that is the gist of student-centered teaching [4, 53]. Allocating resources on concepts that dental students struggle with, particularly when they move from learning basic medical sciences to pre-clinical laboratory work and then into the clinic environment will improve learning. The first-time students feel “stuck” and unsure of their knowledge are frequently the transitional phases where they meet real patients with a variety of unique clinical presentations and issues, combined with varying anatomical features. At this point, TC identification in conjunction with pertinent reflective discussion and assessment exercises may help students cross these phases more easily [24]. TCs can provide students with roadmaps and standards as they navigate through that transitional environment and develop their own scholarly literacy [53]. The TC framework compels educators to reconsider some of their conventional beliefs regarding andragogy. Most importantly, the intricacy and diversity of each learner’s transformation (comprehension, experimentation, and reflection on integration) pose a challenge from linear learning to outcome-based curriculum models and also demand a critical approach towards our perception of spiral curriculum. Threshold concepts can be helpful to explore how we teach ways of thinking and practicing, grasp the interaction between formal and hidden curriculum, and provide students with new perspectives on learning [5].

While some educators insist on the necessity of constructing a solid orthodontic theoretical background and developing practical experience, others state that undergraduate dental students should be provided with only the fundamental knowledge [54]. Following three iterative rounds of Delphi, the current investigation revealed the following 34 threshold concepts. The study involved three types of concepts as described by Davies and Mangan model. The list of concepts included basic concepts of orthodontics like growth and development, occlusion, mandibular rotations, growth pattern of mandible and maxilla. Then comes the procedural concepts which involves skills component of the undergraduate orthodontic curriculum i.e., patient assessment, analysis of diagnostic records and orthodontic appliances. Disciplinary concepts, involve the ways of thinking in the discipline i.e., history taking of orthodontic patient, ethical practice, diagnosis and treatment planning etc. This requires not only the understanding of concept but also involves organization of ones thinking and thus transformation. So, we can say that these three types of concepts in a discipline can have characteristics (transformative, troublesome, integrative, irreversible & bounded) as described by Meyer and Land. While Davies and Mangan’s model delves into the concept’s application, Meyer and Land’s model clarifies the concept’s appearance in terms of their criteria. Combining them gives a more comprehensive image of what a threshold concept might entail [31].

Previous studies gave an overview of the orthodontic curriculum and the skills that undergraduates should or should not be able to employ, but they fail to discuss any TCs particular to the field of orthodontics. The educators agreed that all undergraduate students need to demonstrate skills such as radiographic interpretation, cephalography, OPG, history taking, oral examination, study casts, and use of removable appliances [1]. Other studies countered that although undergraduate education should teach these skills in theory, practice should follow, possibly during a clinical internship or orthodontic residency [55]. Green and Rasmussen necessitated students to overcome four TCs in order to succeed as dentists. i.e., dealing with the whole patient, you may not know everything, accountability, problem-solving, and adaptability—are necessary for students to successfully complete their dental education [56]. Malocclusion, interceptive orthodontics and when to refer patients for specialized treatment, were unanimously pointed as TCs during this study as indicated by past studies [57]. The placement of brackets on a typodont was recommended for practice, but the prescription of brackets was considered beyond the level of undergraduates.

The experts recommended demonstrations and interactive lectures for knowledge-related components, and their assessment was agreed upon through MCQs and SAQs. Practical exercises using patient diagnostic records, role plays, clinical clerkships/chairside teaching, and SGDs were generally agreed upon for skill and attitude-related TCs. These domains should be assessed using MCQs, OSCE, TOACS, DOPS, and Viva Voce. The objective of assessment is to provide students with the chance to exhibit their subject-matter expertise by utilizing tailored academic literacies or attributes. The two must be interconnected in order to effectively develop curricula and offer transition support [53]. Medical schools reported the assessment of undergraduate students in clinical and laboratory settings as well as the use of objective structured clinical examinations (OSCEs) and written tests in orthodontics. Appropriate assessment methods were deemed necessary [1]. Prior research indicated that students utilized interactive online courses centered around many threshold concepts. Other strategies included substituting role plays for traditional teaching and reading other students’ remarks on online discussion boards about specific threshold topics, which exposed them to a variety of perspectives [52]. A learner-centered strategy the flipped classroom was identified as a valuable approach to teaching TCs in physiology [58].

Clinical clerkships and flipped classrooms were not suggested for the TCs in the domains of growth and development as well as recent advances. Interactive lectures were considered unnecessary for TCs pertaining to patient assessment and diagnosis. SEQs was the least selected assessment method. Many discussions regarding TCs revolves around transformative learning. This transformation occurs not only at cognitive level but is also related to Affective and psychomotor domains [17]. By identifying TCs we attempted to streamline the content to be taught/learning outcomes in undergraduate orthodontic curriculum, in a manner beneficial to both educators and learners [8].

### Limitations

This study had some limitations.

1. TCs in itself was a difficult concept to grasp for the participants and the work done in the respective field was negligible or might be out of our access to build up a good discussion.
2. Our current work focused on identifying TCs and proposed teaching and assessment methods for these, it did not include determining the nature of these TCs.
3. The study encompassed the extensive undergraduate curriculum, some of the TCs may have been neglected. The questionnaire format was modified in each round to ensure optimization and avoid respondent’s fatigue or disinterest.
4. Phenomena like belief perseverance and respondents’ fatigue and attrition were observed as participants liked to stick to their initial responses and were reluctant to address the same questions again [59].

### Future implication

Future research should focus on integrating these concepts into undergraduate orthodontic curriculum and implementing teaching and assessment strategies within the discipline to enhance dental education across institutions and evaluate their impact on student performance and learning. Introduction/development of innovative instruction techniques to help students cross these thresholds can be investigated further. Furthermore, TCs for postgraduate orthodontic trainees needs to be identified.

## Conclusion

The study resulted in identification of 34 TCs in orthodontics and their respective TS and AS. Many of these strategies are already in use in different institutes across country. The findings of this study can help focus on key areas that may alter learner’s perspectives and reduce cognitive overload in educational settings. Appropriate teaching and learning techniques are essential due to clinical nature of the discipline which necessitates patient interaction and application of theoretical knowledge in clinical context to effectively close the theory-practice gap. Educators must recognize this integral aspect of learning and provide opportunities for students to overcome these challenges, enabling them to think, communicate and perform as experts.

## Supplementary Information

Supplementary Material 1.

Supplementary Material 2.

Supplementary Material 3.

## Data Availability

The datasets used or analyzed during the current study are available from the corresponding author on reasonable request.

## Abbreviations

AS: Assessment Strategies
CBL: Case Based Learning
CREDES: Guidance on Conducting and Reporting Delphi Studies
CA: Consensus achieved
CNA: Consensus not achieved
DOPS: Direct observation of practical skills
MCPS: Member of College of Physicians and Surgeons Pakistan
MCQ: Multiple Choice Questions
MDS: Master of Dental Surgery
MHPE: Masters in Health Professional Education
Mini-CEX: Mini-Clinical Evaluation Exercise
MOrth: Membership in Orthodontics
OSCE: Objectively Structured Clinical Examination
PAO: Pakistan Association for Orthodontics
PMDC: Pakistan Medical & Dental Council
SAQs: Short answer question
SEQs: Short essay questions
STEM: Science, Technology, Engineering, and Mathematics
TCF: Threshold Concept Framework
TCs: Threshold Concepts
TOACS: Task-Oriented Assessment of Clinical Skills
TS: Teaching Strategies
