# Supplementary material for: Identification of threshold concepts in the undergraduate orthodontics curriculum: a modified Delphi study
Source: BMC Med Educ. 2026 Jan 9;26:213. doi: 10.1186/s12909-025-08516-6 (PMC12882480; doi:10.1186/s12909-025-08516-6)
Supplement: Supplementary file 1 — Supplementary Material 1. [file 12909_2025_8516_MOESM1_ESM.docx]

**Annexure I-** **Questions for Focus Group Discussions (FGDs)**

**FGD 1&3- Identification of Threshold concepts in undergraduate orthodontics curriculum.**

Q1. In your opinion what were the most challenging/transformative concepts for your students in

orthodontics? OR

What topics did you find challenging to understand in orthodontics undergraduate curriculum?

(Keeping in mind 5 characteristics of threshold concepts)

Q2. What concepts as per your understanding/experience should have been made clear to you in

this course or perhaps earlier in your education?

Q3. Are there any concepts that you have learned in this area that have really changed your

outlook?

**FGD 2- Teaching strategies for Threshold Concepts in orthodontics undergraduate curriculum**

Q4. How will you assist/ teach these concepts to your students in orthodontics at undergraduate

level? OR

What are Teaching strategies/new innovative approaches that you think are appropriate or you

are using that will help students crossing these thresholds?

**Assessment of Threshold Concepts in orthodontics undergraduate curriculum**

Q5. How can you determine if students have understood these threshold concept and what

methods can you use to assess their understanding? OR

How would you assess that the concepts have been fully understood and internalized?

**Annexure II- Modified Delphi Round I Questionnaire**

Title of the study: **Identification of threshold concepts in orthodontics’ undergraduate dental curriculum-A modified Delphi study**

**Researcher: Dr Aisha Sher**

Demonstrator, Medical Education Department

Margalla College of Dentistry,

Email: [dr.aisha_sher@yahoo.com](mailto:dr.aisha_sher@yahoo.com)

Mobile# 03314947185

**Respected Sir/Madam!**

I'm inviting you to participate in a study as part of my Masters in Health Profession Education (MHPE) thesis from Riphah International university. The study aims to:

1. ***Identify Threshold concepts in the orthodontic undergraduate dental curriculum.***
2. ***Determine teaching and assessment strategies of TCs in the orthodontic undergraduate curriculum.***

The intent behind identification of threshold concepts is to improve the learning experience of students and avoid stuffing of curriculum.

Regards,

Dr. Aisha Sher.

**INTRODUCTION:**

Orthodontics involves anatomy and physiology, growth and development, biomechanics, mechanical mechanics and other aspects, so it is difficult to learn and master such a large and complex theoretical knowledge and its practical application.

**Threshold concepts (TCs), first suggested by Meyer and Land in 2003, are likened to portals because once a learner grasps and comprehends these challenging ideas, they begin to think differently about a discipline. (J. A. N. H. F. Meyer & Land, 2005). Threshold concepts are key to achieving mastery of a subject.**

There are five key characteristics of a Threshold Concept:

1. ***Transformative*,** it transforms the way a learner thinks about the discipline. As a result, the learner develops a new sense of self-identification and a fresh perspective on their role in the new profession.

**2. *Irreversible*,** as it is unlikely that newly acquired information would be forgotten or unlearned with much difficulty after it has been accepted.

**3. *Integrative****,* understanding of the threshold concept makes new links with other topics of the subject.

4**. *Bounded****,* threshold concepts are unique to the discipline and helps to define its boundaries

**5. *Troublesome***, knowledge which is complex to understand., Alien (emanating from another culture or discourse), or seemingly incoherent' (cousin, 2006); students may have difficulty coping with the new perspective that is offered.

**DATA COLLECTION PROCEDURE:**

A modified Delphi study design with three iterative rounds is being employed to reach an expert consensus. 35 potential threshold concepts are proposed under 9 domains of orthodontics at undergraduate level, after a number of multi-level focus group discussions. This online questionnaire includes 35 items to develop a consensus if they possess characteristics of a threshold concept on a 3-point Likert scale.

**INSTRUCTIONS FOR FILLING THE QUESTIONNAIRE:**

(Please only continue after reading the literature and viewing the attached presentation on threshold concepts)

The first round of Delphi is currently underway. During the first round, consensus will be built if the proposed concepts serve as threshold concepts.

This questionnaire contains 35 proposed concepts, each of which is followed by a 3-point Likert scale**. To qualify as a threshold concept, an item must possess at least two out of five characteristics of a threshold concept listed above. One of these characteristics should either be Transformative or Troublesome.**

For your valuable suggestions, comments section have been added at the end of the questionnaire.

The questionnaire will require about 15-20 minutes of your valuable time to complete. I will be highly obliged for this favor.

**INFORMED CONSENT:**

- You have been chosen for this study because of your orthodontic knowledge and teaching experience.
- Your participation in this study is highly appreciated and is completely voluntary.
- Your personal information and responses to the questionnaire will be kept highly confidential.
- If you have any questions or need any additional information, please feel free to contact, phone number and email address is provided.

**PARTICIPANT CERTIFICATION:**

I understand the overall aim and research methodology of this study. I am assured that anonymity and confidentiality will be maintained. By filling out this form, I therefore give my consent to participate.

**PARTICIPANT’S INFORMATION:**

Name: _______________________________________________

Designation: ___________________________________________

Qualification (Post Graduate): _____________________________

Teaching Experience (in years): ____________________________

Institute and City: ______________________________________

Mobile number: _______________________________________

Email address: ________________________________________

| **Sr#** | **Domains** | **Proposed Threshold Concepts** | **Agreement** |
| --- | --- | --- | --- |
| I | **Patient’s information** | 1.Taking history of Orthodontic patient | Agree/ indecisive /disagree |
| II | **Growth and development** | 2.Growth pattern of Mandible and Maxilla  3.Significance of mandibular rotations  4.Various growth indicators determining the peak skeletal growth/Growth Assessment parameters | Agree/ indecisive /disagree  Agree/ indecisive /disagree  Agree/ indecisive /disagree |
| III | **Patient’s Clinical Evaluation** | 5.Soft tissue paradigm  6. Facial profile analysis | Agree/ indecisive /disagree  Agree/ indecisive /disagree |
| IV | **Analysis of diagnostic records** | 7.Arch dimensions (Arch length, width, depth & Curve of Wilson, Spee, Monson)  8.Eyeballing method of calculating ALD  9.Bolton’s analysis  10.OPG analysis/Dental age analysis of OPG  11.Analysis of CVM stages on lateral Cephalogram  12.Limitations of ANB angle  13.Composite Mixed dentition analysis | Agree/ indecisive /disagree  Agree/ indecisive /disagree  Agree/ indecisive /disagree  Agree/ indecisive /disagree  Agree/ indecisive /disagree  Agree/ indecisive /disagree  Agree/ indecisive /disagree |
| V | **Diagnosis** | 14.Making a problem list  15.Diagnosis of growth and developmental disorders  16.Diagnosis of impacted maxillary canines. | Agree/ indecisive /disagree  Agree/ indecisive /disagree  Agree/ indecisive /disagree |
| VI | **Treatment of orthodontic patient- Basic concepts and goals** | 17.Treatment planning of various Malocclusions  18.Significance of molar (healthy/mutilated) relationship  19.Extraction patterns (molar/premolar/Incisor) in treatment planning  20. Balancing and compensating extractions of 6s.  21.Periodontal considerations for orthodontic treatment  22.Basics of biomechanics  23.Basics of Anchorage control in orthodontics  24.Retention and Relapse  25.Clinical Implications of developmental disorders in orthodontics  26.Knowing when and whom to refer  27.Iatrogenic effects of orthodontic treatment | Agree/ indecisive /disagree  Agree/ indecisive /disagree  Agree/ indecisive /disagree  Agree/ indecisive /disagree  Agree/ indecisive /disagree  Agree/ indecisive /disagree  Agree/ indecisive /disagree  Agree/ indecisive /disagree  Agree/ indecisive /disagree  Agree/ indecisive /disagree  Agree/ indecisive /disagree |
| VII | **Interceptive orthodontics** | 28.Management of 1^st^ molars with poor prognosis  29.Interception of incisor Trauma  30.Mixed dentition space management protocols (space maintenance/regaining, supervision/serial extraction) | Agree/ indecisive /disagree  Agree/ indecisive /disagree  Agree/ indecisive /disagree |
| VIII | **Appliances** | 31.Indications of various appliances (Removable/functional/fixed)  32.Bracket placement  33.Prescriptions of brackets | Agree/ indecisive /disagree  Agree/ indecisive /disagree  Agree/ indecisive /disagree |
| IX | **Recent Advances** | 34.Concept of clear aligners  35.Basic concept of CBCT | Agree/ indecisive /disagree  Agree/ indecisive /disagree |

This is a short list of threshold concepts that have been put forth in the undergraduate orthodontic curriculum. Please suggest any other concepts that you believe meet the criteria for being designated as "threshold concept” or if some modifications are required:

**______________________________________________________________________________________________________________________________________**

Please suggest appropriate teaching strategies for threshold concepts to improve learning of students: ____________________________________________________________________________________________________________________________________________________

Please suggest Assessment methods for these threshold concepts: ______________________________________________________________________________________________________________________________________________________________________________________________________________________________

**Annexure III- Modified Delphi Round 2 Questionnaire**

**Identification of Threshold Concepts in Undergraduate Orthodontics' Curriculum-A Modified Delphi Study.**

**Section-1**

**Researcher: Dr. Aisha Sher**

Demonstrator, Medical Education Department,

Margalla College of Dentistry, Rawalpindi.

Email: [dr.aisha_sher@yahoo.com](mailto:dr.aisha_sher@yahoo.com)

Mobile# 03314947185

**Respected Sir/Madam!**

I'm inviting you to participate in a study as part of my Masters in Health Profession Education (MHPE) thesis from Riphah International university. The study aims to:

1. ***Identify Threshold concepts in the orthodontic undergraduate dental curriculum.***

***2. Determine teaching and assessment strategies of TCs in the orthodontic undergraduate curriculum.***

The intent behind identification of threshold concepts is to improve the learning experience of students and avoid stuffing of curriculum.

Regards,

Dr Aisha Sher.

**INTRODUCTION:**

Orthodontics involves anatomy, facial bony structures, growth and development, biomechanics, mechanical principles and other aspects, so it is difficult to learn and master such a large and complex theoretical knowledge and its practical application.

**Threshold concepts (TCs), first suggested by Meyer and Land in 2003, are likened to portals because once a learner grasps and comprehends these challenging ideas, they begin to think differently about a discipline. (J. A. N. H. F. Meyer & Land, 2005). Threshold concepts are key to achieving mastery of a subject.**

There are five key characteristics of a Threshold Concept:

**1.** ***Transformative*,** it transforms the way a learner thinks about the discipline. As a result, the learner develops a new sense of self-identification and a fresh perspective on their role in the new profession.

**2. *Irreversible*,** as it is unlikely that newly acquired information would be forgotten or unlearned with much difficulty after it has been accepted.

**3. *Integrative****,*understanding of the threshold concept makes new links with other topics of the subject.

**4. *Bounded****,* threshold concepts are unique to the discipline and helps to define its boundaries.

***5. Troublesome***, knowledge which is complex to understand., Alien (emanating from another culture or discourse) or seemingly incoherent' (cousin, 2006); students may have difficulty coping with the new perspective that is offered.

**INSTRUCTIONS FOR FILLING THE QUESTIONNAIRE:**

(**Dear participant, the results of Round 1 are shared separately with you, you can reconsider your answers)**

This is the **Round 2**of the Modified Delphi study. **During the Round 1, agreement has been achieved on 27 out of 35 proposed concepts. These 27 concepts are added separately in questionnaire section 2, to assess the stability of responses. If you want to reconsider/change some/all of your responses from round 1 you can fill in Section 2. If your responses are same as round 1 after reviewing the document attached you can proceed to the next section.**

The **section 3**of this questionnaire contains **8 items from Round 1 and 4 new items suggested by experts**. To reconsider responses from round 1 you can refer to group consensus which is shared with you as a word document. You can state the reason to change your response at the end of section.

The **section 4**comprises of **teaching and assessment strategies** to teach and assess the proposed TCs. These are based on expert opinions from Round 1 and prior Focus group discussions.

**To qualify as a threshold concept, an item must possess at least two out of five characteristics of a threshold concept listed above. One of these characteristics should either be Transformative or Troublesome.**

The questionnaire will take about **10-15 minutes** of your valuable time to complete. **I will be highly obliged for this favor.**

**PARTICIPANT CERTIFICATION:**

I understand the overall aim and research methodology of this study. I am assured that anonymity and confidentiality will be maintained. By filling out this form, I therefore give my consent to participate.

Name: _______________________________________________

Designation: ___________________________________________

Qualification (Post Graduate): _____________________________

Teaching Experience (in years): ____________________________

Institute and City: ______________________________________

Mobile number: _______________________________________

Email address: ________________________________________

**Section 2-Threshold Concepts that reached consensus in round 1.**

This section contains 27 Threshold concepts on which consensus has been achieved in Round 1 **(80% or more experts agree that these are TCs).**

| **Sr#** | **Domains** | **Proposed Threshold Concepts** | **Agreement** |
| --- | --- | --- | --- |
| I | **Patient’s information** | 1.Taking history of Orthodontic patient | Agree/ indecisive /disagree |
| II | **Growth and development** | 2.Growth pattern of Mandible and Maxilla  3.Significance of mandibular rotations  4.Various growth indicators determining the peak skeletal growth/Growth Assessment parameters | Agree/ indecisive /disagree  Agree/ indecisive /disagree  Agree/ indecisive /disagree |
| III | **Patient’s Clinical Evaluation** | 5.Soft tissue paradigm  6. Facial profile analysis | Agree/ indecisive /disagree  Agree/ indecisive /disagree |
| IV | **Analysis of diagnostic records** | 7.Arch dimensions (Arch length, width, depth & Curve of Wilson, Spee, Monson)  8.OPG analysis/Dental age analysis of OPG  9.Analysis of CVM stages on lateral Cephalogram | Agree/ indecisive /disagree  Agree/ indecisive /disagree  Agree/ indecisive /disagree |
| V | **Diagnosis** | 10.Making a problem list  11.Diagnosis of growth and developmental disorders  12.Diagnosis of impacted maxillary canines. | Agree/ indecisive /disagree  Agree/ indecisive /disagree  Agree/ indecisive /disagree |
| VI | **Treatment of orthodontic patient- Basic concepts and goals** | 13.Treatment planning of various Malocclusions  14.Significance of molar (healthy/mutilated) relationship  15.Extraction patterns (molar/premolar/Incisor) in treatment planning  16.Periodontal considerations for orthodontic treatment  17.Basics of biomechanics  18.Basics of Anchorage control in orthodontics  19.Retention and Relapse  20.Clinical Implications of developmental disorders in orthodontics  21.Knowing when and whom to refer  22.Iatrogenic effects of orthodontic treatment | Agree/ indecisive /disagree  Agree/ indecisive /disagree  Agree/ indecisive /disagree  Agree/ indecisive /disagree  Agree/ indecisive /disagree  Agree/ indecisive /disagree  Agree/ indecisive /disagree  Agree/ indecisive /disagree  Agree/ indecisive /disagree  Agree/ indecisive /disagree |
| VII | **Interceptive orthodontics** | 23.Management of 1^st^ molars with poor prognosis  24.Interception of incisor Trauma  25.Mixed dentition space management protocols (space maintenance/regaining, supervision/serial extraction) | Agree/ indecisive /disagree  Agree/ indecisive /disagree  Agree/ indecisive /disagree |
| VIII | **Appliances** | 26.Indications of various appliances (Removable/functional/fixed)  32.Bracket placement  33.Prescriptions of brackets | Agree/ indecisive /disagree  Agree/ indecisive /disagree  Agree/ indecisive /disagree |
| IX | **Recent Advances** | 27.Basic concept of CBCT | Agree/ indecisive /disagree |

**Section 3**: **Proposed Threshold concepts that didn't reach consensus in Round 1.**

This section contains 8 concepts from Round 1 and 4 newly proposed TCs. You can re-consider your prior response in accordance with group agreement.

| **Domains** | **Proposed Threshold Concepts** | **Agreement** |
| --- | --- | --- |
| **Analysis of diagnostic records** | 1.Eyeballing method of calculating ALD | Agree/ indecisive /disagree |
|  | 2.Bolton’s analysis | Agree/ indecisive /disagree |
|  | 3.Limitations of ANB angle | Agree/ indecisive /disagree |
|  | 4.Mixed dentition analysis | Agree/ indecisive /disagree |
| **Treatment of orthodontic patient- Basic concepts and goals** | 5. Balancing and compensating extractions | Agree/ indecisive /disagree |
| **Appliances** | 6.Bracket placement | Agree/ indecisive /disagree |
|  | 7.Prescriptions of brackets | Agree/ indecisive /disagree |
| **Recent Advances** | 8.Concept of clear aligners | Agree/ indecisive /disagree |
| **Concepts Suggested by Experts in Round 1** | 9. Occlusion | Agree/ indecisive /disagree |
|  | 10.Etiology of Malocclusion | Agree/ indecisive /disagree |
|  | 11.Biology of tooth movement | Agree/ indecisive /disagree |
|  | 12.Ethical practice | Agree/ indecisive /disagree |

**Section 4: Teaching strategies that can assist students grasp these TCs & Assessment strategies that can assess student understanding of these concepts.**

The 9 domains from Round 1 are combined under 5 broader Categories for the ease of participants.

| **Domains & Strategies** | | **Agreement** | | |
| --- | --- | --- | --- | --- |
|  |  | **Agree** | **Indecisive** | **Disagree** |
| **I-Patient’s information**  **II- Patient’s Clinical Evaluation**   - Taking history of Orthodontic patient - Soft tissue paradigm - Facial profile analysis | | | | |
| **Teaching Strategies** | 1.Clinical clerkship/Chair side teaching |  |  |  |
|  | 2.SGD (simulated patients, Role play, tutorial, CBL) |  |  |  |
|  | 3. Demonstration |  |  |  |
|  | 4. Case presentation |  |  |  |
|  | 5.Interactive lecture |  |  |  |
| **Assessment Strategies** | 1.MCQs |  |  |  |
|  | 2.SEQs |  |  |  |
|  | 3.Viva Voce |  |  |  |
|  | 4.Mini-CEX |  |  |  |
|  | 5.OSCE |  |  |  |
|  | 6.TOACS |  |  |  |
| **III- Growth and development**   - Growth pattern of Mandible and Maxilla - Significance of mandibular rotations - Various growth indicators determining the peak skeletal growth/Growth Assessment parameters - Occlusion - Biology of tooth movement - Etiology of Malocclusion | | | | |
| **Teaching Strategies** | 1.Interactive Lecture |  |  |  |
|  | 2.SGD (Tutorials, CBL) |  |  |  |
|  | 3.Demonstrations |  |  |  |
|  | 4.Clinical Clerkship (Assessment of Patient's Orthodontic records) |  |  |  |
|  | 5.Flipped Classroom |  |  |  |
| **Assessment Strategies** | 1.MCQs |  |  |  |
|  | 2.SAQs |  |  |  |
|  | 3.SEQs |  |  |  |
|  | 4.OSCE |  |  |  |
|  | 5.Viva Voce |  |  |  |
| **IV- Analysis of diagnostic records**  **V- Diagnosis**   - Arch dimensions (Arch length, width, depth & Curve of Wilson, Spee, Monson) - Eyeballing method of calculating ALD - Bolton’s analysis - OPG analysis/Dental age analysis of OPG - Analysis of CVM stages on lateral Cephalogram - Limitations of ANB angle - Mixed dentition analysis - Making a problem list - Diagnosis of growth and developmental disorders - Diagnosis of impacted maxillary canines | | | | |
| **Teaching Strategies** | 1.Interactive Lecture |  |  |  |
|  | 2.SGD (Tutorials, CBL) |  |  |  |
|  | 3.Demonstrations |  |  |  |
|  | 4.Hands on activities (Assessment of Patient's Orthodontic records) |  |  |  |
|  | 5.Clinical Clerkship/ Chairside teaching |  |  |  |
|  | 6.Case presentations |  |  |  |
| **Assessment Strategies** | 1.MCQs |  |  |  |
|  | 2.SEQs |  |  |  |
|  | 3.Clinical Targets |  |  |  |
|  | 4.OSCE |  |  |  |
|  | 5.Viva Voce |  |  |  |
|  | 6.TOACS |  |  |  |
| **VI- Treatment of orthodontic patient- Basic concepts and goals**  **VII- Interceptive orthodontics**  **VIII- Appliances**   - Treatment planning of various Malocclusions - Significance of molar (healthy/mutilated) relationship - Extraction patterns (molar/premolar/Incisor) in treatment planning - Balancing and compensating extractions - Periodontal considerations for orthodontic treatment - Basics of biomechanics - Basics of Anchorage control in orthodontics - Basics of Retention and Relapse - Clinical Implications of developmental disorders in orthodontics - Knowing when and whom to refer - Ethical practice - Iatrogenic effects of orthodontic treatment - Management of 1st molars with poor prognosis - Interception of incisor Trauma - Mixed dentition space management protocols (space maintenance/regaining, supervision/serial extraction) - Indications of various appliances (Removable/functional/fixed) - Bracket placement - Prescriptions of brackets | | | | |
| **Teaching Strategies** | 1.Interactive Lecture |  |  |  |
|  | 2.SGD (Tutorials, CBL) |  |  |  |
|  | 3.Demonstrations |  |  |  |
|  | 4.Hands on activities (Assessment of Patient's Orthodontic records) |  |  |  |
|  | 5.Clinical Clerkship/ Chairside teaching |  |  |  |
|  | 6.Case presentations |  |  |  |
| **Assessment Strategies** | 1.MCQs |  |  |  |
|  | 2.SEQs |  |  |  |
|  | 3.SAQs |  |  |  |
|  | 4.OSCE |  |  |  |
|  | 5.Viva Voce |  |  |  |
|  | 6.DOPS |  |  |  |
| **IX- Recent Advances**   - Concept of clear aligners - Basic concept of CBCT | | | | |
| **Teaching Strategies** | 1.Interactive Lecture |  |  |  |
|  | 2.Flipped classroom |  |  |  |
|  | 3.SGD |  |  |  |
|  | 4.Clinical Clerkship/ Chairside teaching |  |  |  |
| **Assessment Strategies** | 1.MCQs |  |  |  |
|  | 2.SAQs |  |  |  |
|  | 3.Viva Voce |  |  |  |

**Suggestions/ Any modification required/ If a particular teaching and assessment strategy should be added for some TCs----------------------------------------------------------------------------------------------------------------------------------------------------------------------------------------------------------------**

**Identification of threshold concepts in orthodontics’ undergraduate dental curriculum-A modified Delphi study (Round 1- Participant Information Sheet)**

Name: _______________________________________________

Designation: ___________________________________________

Qualification (Post Graduate): _____________________________

Teaching Experience (in years): ____________________________

Institute and City: ______________________________________

Mobile number: _______________________________________

Email address: ________________________________________

| **Domains & Proposed Threshold Concepts** | **Your Response** | **Group’s Agreement** | | | **Consensus**  **(CA/CNA)** | **Change response+ Reason** |
| --- | --- | --- | --- | --- | --- | --- |
|  |  | **Agree** | **Indecisive** | **Disagree** |  |  |
| **I Patient’s information** | | | | | | |
| 1.Taking history of Orthodontic patient |  | 39(95.1%) | - | 2(4.9%) | CA-TC |  |
| **II Growth and development** | | | | | | |
| 2.Growth pattern of Mandible and Maxilla  3.Significance of mandibular rotations  4.Various growth indicators determining the peak skeletal growth/Growth Assessment parameters |  | 40(97.6%)  34(82.9%)  38(92.7%) | 1(2.4%)  5(12.2%)  2(4.9%) | -  2(5.9%)  1(2.4%) | CA-TC  CA-TC  CA-TC |  |
| **III Patient’s Clinical Evaluation** | | | | | | |
| 5.Soft tissue paradigm 6. Facial profile analysis |  | 34(82.9%)36(87.8%) | 6(14.6%)  3(7.3%) | 1(2.4%)  2(4.9%) | CA-TC  CA-TC |  |
| **IV Analysis of diagnostic records** | | | | | | |
| 7.Arch dimensions (Arch length, width, depth & Curve of Wilson, Spee, Monson)  8.Eyeballing method of calculating ALD  9.Bolton’s analysis  10.OPG analysis/Dental age analysis of OPG  11.Analysis of CVM stages on lateral Cephalogram  12.Limitations of ANB angle  13.Composite Mixed dentition analysis |  | 34(82.9%)  31(75.6%)  32(78%)  37(90.2%)  36(87.8%)  23(56.1%)  31(75.6%) | 6(14.6%)  3(7.3%)  4(9.8%)  2(4.9%)  3(7.3%)  23(56.1%)  31(75.6%) | 1(2.4%)  7(17.1%)  5(12.2%)  2(4.9%)  2(4.9%)  8(19.5%)  4(9.8%) | CA-TC  CNA  CNA  CA-TC  CA-TC  CNA  CNA |  |
| **V Diagnosis** | | | | | | |
| 14.Making a problem list  15.Diagnosis of growth and developmental disorders  16.Diagnosis of impacted maxillary canines. |  | 36(87.8%)35(85.4%)  36(87.8%) | 3(7.3%)  4(9.8%)  2(4.9%) | 2(4.9%)  2(4.9%)  3(7.3%) | CA-TC  CA-TC  CA-TC |  |
| **VI Treatment of orthodontic patient- Basic concepts and goals** | | | | | | |
| 17.Treatment planning of various Malocclusions  18.Significance of molar (healthy/mutilated) relationship  19.Extraction patterns (molar/premolar/Incisor) in treatment planning  20. Balancing and compensating extractions of 6s.  21.Periodontal considerations for orthodontic treatment  22.Basics of biomechanics  23.Basics of Anchorage control in orthodontics  24.Basics of Retention and Relapse  25.Clinical Implications of developmental disorders in orthodontics  26.Knowing when and whom to refer  27.Iatrogenic effects of orthodontic treatment |  | 35(85.4%)  39(95.1%)  36(87.8%)  29(17.7%)  35(85.4%)  37(90.2%)  39(95.1)  40(97.6%)  34(82.9%)  39(95.1%)  37(90.2%) | 3(7.3%)  2(4.9%)  3(7.3%)  7(17.1%)  2(4.9%)  -  -  1(2.4%)  6(14.6%)  2(4.9%)  2(4.9%) | 3(7.3%)  -  2(4.9%)  5(12.2%)  4(9.8%)  4(9.8%)  2(4.9%)  -  1(2.4%)  -  2(4.9%) | CA-TC  CA-TC  CA-TC  CNA  CA-TC  CA-TC  CA-TC  CA-TC  CA-TC  CA-TC  CA-TC |  |
| **VII Interceptive orthodontics** | | | | | | |
| 28.Management of 1st molars with poor prognosis  29.Interception of incisor Trauma  30.Mixed dentition space management protocols (space maintenance/regaining, supervision/serial extraction) |  | 33(80.5%)  37(90.2%)  34(82.9%) | 6(14.6%)  3(7.3%)  4(9.8%) | 2(4.9%)  1(2.4%)  3(7.3%) | CA-TC  CA-TC  CA-TC |  |
| **VIII Appliances** | | |  |  |  |  |
| 31.Indications of various appliances (Removable/functional/fixed)  32.Bracket placement  33.Prescriptions of brackets |  | 37(90.2%)  28(68.3%)  15(36.6%) | 1(2.4%)  3(7.3%)  11(26.8%) | 3(7.3%)  10(24.4%)  15(36.6%) | CA-TC  CNA  CNA |  |
| **IX Recent Advances** | | | | | | |
| 34.Concept of clear aligners  35.Basic concept of CBCT |  | 27(65.9%)33(80.5%) | 10(24.4%)  5(12.2%) | 4(9.8%)  3(7.3%) | CNA  CA-TC |  |

**Abbreviations: Consensus achieved=CA, Consensus not achieved=CNA, Threshold Concept=TC**

**Annexure IV- Modified Delphi Round 3 Questionnaire**

**Identification of threshold concepts in orthodontics’ undergraduate dental curriculum-A modified Delphi study (Round 2- Questionnaire & Participant Information Sheet)**

**Section-1**

**Researcher: Dr Aisha Sher**

Demonstrator, Medical Education Department,

Margalla College of Dentistry,

Email: [dr.aisha_sher@yahoo.com](mailto:dr.aisha_sher@yahoo.com)

Mobile# 03314947185

**Respected Sir/Madam!**

Thank you for being an integral part of this study. I’m inviting you to participate in the 3rd and Last Round of the study which is a part of my Masters in Health Profession Education (MHPE) thesis. As described earlier the study aims to:

1. ***Identify Threshold concepts in the orthodontic undergraduate dental curriculum.***

***2. Determine teaching and assessment strategies of TCs in the orthodontic undergraduate curriculum.***

The intent behind identification of threshold concepts is to improve the learning experience of students and avoid stuffing of curriculum.

Regards,

Dr Aisha Sher.

**INTRODUCTION:**

**Threshold concepts (TCs), first suggested by Meyer and Land in 2003, are likened to portals because once a learner grasps and comprehends these challenging ideas, they begin to think differently about a discipline. (J. A. N. H. F. Meyer & Land, 2005). Threshold concepts are key to achieving mastery of a subject.**

There are five key characteristics of a Threshold Concept:

**1.** ***Transformative*,** it transforms the way a learner thinks about the discipline. As a result, the learner develops a new sense of self-identification and a fresh perspective on their role in the new profession.

**2. *Irreversible*,** as it is unlikely that newly acquired information would be forgotten or unlearned with much difficulty after it has been accepted.

**3. *Integrative****,*understanding of the threshold concept makes new links with other topics of the subject.

**4. *Bounded****,* threshold concepts are unique to the discipline and helps to define its boundaries.

***5. Troublesome***, knowledge which is complex to understand., Alien (emanating from another culture or discourse) or seemingly incoherent' (cousin, 2006); students may have difficulty coping with the new perspective that is offered.

**INFORMATION & INSTRUCTIONS:**

(**Dear participant, the results of Round 2 and questionnaire are shared separately with you as a word document, please review them before filling this questionnaire)**

During Round 2 consensus has been achieved on 7 out of 12 proposed TCs. Consensus has also been achieved on majority of Teaching and Assessment Strategies for TCs included in Round 2 under 9 Domains of orthodontics.

For your convenience all the items are**also send separately as a word document along with group agreement, to determine the stability of prior responses.**

This form has 5 TCs and few Teaching and Assessment Strategies on which consensus has not been achieved. Please reconsider your responses with reference to group agreement. **The items achieving 80% agreement will be retained as TCs and Appropriate Teaching and Assessment strategies while others will be excluded from the list. However, to change your prior responses on which agreement has already been achieved please refer to word document, edit and send it back.**

**To qualify as a threshold concept, an item must possess at least two out of five characteristics of a threshold concept listed above. One of these characteristics should either be Transformative or Troublesome.**

**Section-2**

**INFORMED CONSENT:**

- You have been chosen for this study because of your orthodontic knowledge and teaching experience.
- Your participation in this study is highly appreciated and is completely voluntary.
- Your personal information and responses to the questionnaire will be kept highly confidential.
- If you have any questions or need any additional information, please feel free to contact, phone number and email address is provided.

**PARTICIPANT CERTIFICATION:**

I understand the overall aim and research methodology of this study. I am assured that anonymity and confidentiality will be maintained. By filling out this form, I therefore give my consent to participate.

Name: _______________________________________________

Designation: ___________________________________________

Qualification (Post Graduate): _____________________________

Teaching Experience (in years): ____________________________

Institute and City: ______________________________________

Mobile number: _______________________________________

Email address: ________________________________________

**Section-3**

**Items with Achieved Consensus***Your responses and group agreement for items on which consensus has been achieved in Round 2 are shared as a word document to check the stability of responses. Please review the attached file before proceeding.

**If you want to change your prior responses for items on which 80% agreement has already been achieved**

YES- Please make changes on the attached word document and send it back.

NO- Your responses are same for items with achieved consensus in round 3. Proceed to the next section.

**Section-4**

**Proposed Threshold concepts that didn't reach consensus in Round 2.**

This section contains 5 items from Round 2. You can re-consider your prior response in accordance with the group agreement.

Abbreviations: Threshold Concepts=TCs, Teaching Strategies=TS, Assessment Strategies=AS, Consensus Achieved=CA, Consensus Not Achieved=CNA

| **Domains & Proposed Threshold Concepts** | **Agreement** | | |
| --- | --- | --- | --- |
|  | **Agree** | **Indecisive** | **Disagree** |
| **Analysis of diagnostic records** | | | |
| 1.Eyeballing method of calculating ALD |  |  |  |
| 3.Limitations of ANB angle |  |  |  |
| **Appliances** | | | |
| 6.Bracket placement  7.Prescriptions of brackets |  |  |  |
| **Recent Advances** | | | |
| 8.Concept of clear aligners |  |  |  |

**TEACHING AND ASSESSMENT**

| **Domains & Strategies** | | **Group Agreement** | | |
| --- | --- | --- | --- | --- |
|  |  | **Agree** | **Indecisive** | **Disagree** |
| **I-Patient’s information**  **II- Patient’s Clinical Evaluation**   - Taking history of Orthodontic patient - Soft tissue paradigm - Facial profile analysis | | | | |
| **TS** | 5.Interactive lecture |  |  |  |
| **AS** | 1.MCQs |  |  |  |
|  | 2.SEQs |  |  |  |
|  | 3.Viva Voce |  |  |  |
|  | 4.Mini-CEX |  |  |  |
| **III- Growth and development**   - **Growth pattern of Mandible and Maxilla** - **Significance of mandibular rotations** - **Various growth indicators determining the peak skeletal growth/Growth Assessment parameters** - **Occlusion** - **Biology of tooth movement** - **Etiology of Malocclusion** | | | | |
| **TS**  **AS** | 3.Demonstrations |  |  |  |
|  | 4.Clinical Clerkship (Assessment of Patient's Orthodontic records) |  |  |  |
|  | 5.Flipped Classroom |  |  |  |
|  | 3.SEQs |  |  |  |
| **IV- Analysis of diagnostic records**  **V- Diagnosis**   - Arch dimensions (Arch length, width, depth & Curve of Wilson, Spee, Monson) - Eyeballing method of calculating ALD - Bolton’s analysis - OPG analysis/Dental age analysis of OPG - Analysis of CVM stages on lateral Cephalogram - Limitations of ANB angle - Mixed dentition analysis - Making a problem list - Diagnosis of growth and developmental disorders - Diagnosis of impacted maxillary canines | | | | |
| **TS** | 1.Interactive Lecture |  |  |  |
| **AS** | 2.SEQs |  |  |  |
| **VI- Treatment of orthodontic patient- Basic concepts and goals**  **VII- Interceptive orthodontics**  **VIII- Appliances**   - Treatment planning of various Malocclusions - Significance of molar (healthy/mutilated) relationship - Extraction patterns (molar/premolar/Incisor) in treatment planning - Balancing and compensating extractions - Periodontal considerations for orthodontic treatment - Basics of biomechanics - Basics of Anchorage control in orthodontics - Basics of Retention and Relapse - Clinical Implications of developmental disorders in orthodontics - Knowing when and whom to refer - Ethical practice - Iatrogenic effects of orthodontic treatment - Management of 1st molars with poor prognosis - Interception of incisor Trauma - Mixed dentition space management protocols (space maintenance/regaining, supervision/serial extraction) - Indications of various appliances (Removable/functional/fixed) - Bracket placement - Prescriptions of brackets | | | | |
| **AS** | 2.SEQs |  |  |  |
| **IX- Recent Advances**   - **Concept of clear aligners** - **Basic concept of CBCT** | | | | |
| **TS**  **AS** | 2.Flipped classroom |  |  |  |
|  | 4.Clinical Clerkship/ Chairside teaching |  |  |  |
|  | 2.SAQs |  |  |  |
|  | 3.Viva Voce |  |  |  |

Comments/Suggestions:----------------------------------------------------------------------------------------------------------------------------------------------------------------------------------------------------------------------------------------------------------------------------------------------

***Attached word document**

**Identification of threshold concepts in orthodontics’ undergraduate dental curriculum-A modified Delphi study (Round 2- Participant Information Sheet)**

Name: _______________________________________________

Designation: ___________________________________________

Qualification (Post Graduate): _____________________________

Teaching Experience (in years): ____________________________

Institute and City: ______________________________________

Mobile number: _______________________________________

Email address: ________________________________________

| **Domains & Proposed Threshold Concepts** | **Your Response** | **Group’s Agreement** | | | **Consensus**  **(CA/CNA)** | **Change in Response+ Reason** |
| --- | --- | --- | --- | --- | --- | --- |
|  |  | **Agree** | **Indecisive** | **Disagree** |  |  |
| **Analysis of diagnostic records** | | | | | | |
| 1.Eyeballing method of calculating ALD  2.Bolton’s analysis |  | 68.4%  84.2% | 10.5%  7.9% | 21.1%  7.9% | CNA  CA-TC |  |
| 3.Limitations of ANB angle  4.Mixed dentition analysis |  | 71.1%  92.1% | 7.9%  2.6% | 21.1%  5.3% | CNA  CA-TC |  |
| **Treatment of orthodontic patient- Basic concepts and goals** | | | | | | |
| 5. Balancing and compensating extractions. |  | 86.8% | 10.5% | 2.6% | CA-TC |  |
| **Appliances** | | | | | | |
| 6.Bracket placement  7.Prescriptions of brackets |  | 65.8%  34.2% | 5.3%  18.4% | 28.9%  36.6% | CNA  CNA |  |
| **Recent Advances** | | | | | | |
| 8.Concept of clear aligners |  | 71.1% | 13.2% | 15.8% | CNA |  |
| **Concepts Suggested by Experts in Round 1** | | | | | | |
| 9. Occlusion |  | 97.4% | - | 2.6% | CA-TC |  |
| 10.Etiology of Malocclusion |  | 92.1% | 2.6% | 5.3% | CA-TC |  |
| 11.Biology of tooth movement |  | 92.1% | 5.3% | 2.6% | CA-TC |  |
| 12.Ethical practice |  | 92.1% | 2.6% | 5.3% | CA-TC |  |

**Abbreviations: Consensus achieved=CA, Consensus not achieved=CNA, Threshold Concept=TC**

**TEACHING AND ASSESSMENT STRATEGIES**

| **Domains & Strategies** | | **Your Response** | **Group Agreement** | | | **Consensus**  **(CA-CNA)** | **Change in response+ Reason** |
| --- | --- | --- | --- | --- | --- | --- | --- |
|  |  |  | **Agree** | **Indecisive** | **Disagree** |  |  |
| **I-Patient’s information**  **II- Patient’s Clinical Evaluation** | | | | | | | |
| **Teaching Strategies** | 1.Clinical clerkship/Chair side teaching |  | 97.4% | - | 2.6% | CA |  |
|  | 2.SGD (simulated patients, Role play, tutorial, CBL) |  | 97.4% | 2.6% | - | CA |  |
|  | 3. Demonstration |  | 92.1% | 2.6% | 5.3% | CA |  |
|  | 4. Case presentation |  | 92.1% | 5.3% | 2.6% | CA |  |
|  | 5.Interactive lecture |  | 78.9% | 13.2% | 7.9% | CNA |  |
| **Assessment Strategies** | 1.MCQs |  | 73.7% | 7.9% | 18.4% | CNA |  |
|  | 2.SEQs |  | 44.7% | 23.7% | 31.6% | CNA |  |
|  | 3.Viva Voce |  | 78.9% | 10.5% | 10.5% | CNA |  |
|  | 4.Mini-CEX |  | 76.3% | 10.5% | 13.2% | CNA |  |
|  | 5.OSCE |  | 94.7% | 2.6% | 2.6% | CA |  |
|  | 6.TOACS |  | 92.1% | 2.6% | 5.3% | CA |  |
| **III- Growth and development** | | | | | | | |
| **Teaching Strategies** | 1.Interactive Lecture |  | 97.4% | - | 2.6% | CA |  |
|  | 2.SGD (Tutorials, CBL) |  | 92.1% | 5.3% | 2.6% | CA |  |
|  | 3.Demonstrations |  | 63.2% | 23.7% | 13.2% | CNA |  |
|  | 4.Clinical Clerkship (Assessment of Patient's Orthodontic records) |  | 76.3% | 7.9% | 15.8% | CNA |  |
|  | 5.Flipped Classroom |  | 73.7% | 18.4% | 7.9% | CNA |  |
| **Assessment Strategies**  **Assessment Strategies** | 1.MCQs |  | 92.1% | 5.3% | 2.6% | CA |  |
|  | 2.SAQs |  | 81.6% | 7.9% | 10.5% | CA |  |
|  | 3.SEQs |  | 65.8% | 7.9% | 26.3% | CNA |  |
|  | 4.OSCE |  | 81.6% | 5.3% | 13.2% | CA |  |
|  | 5.Viva Voce |  | 86.8% | 7.9% | 5.3% | CA |  |
| **IV- Analysis of diagnostic records**  **V- Diagnosis** | | | | | | | |
| **Teaching Strategies** | 1.Interactive Lecture |  | 71.1% | 10.5% | 18.4% | CNA |  |
|  | 2.SGD (Tutorials, CBL) |  | 92.1% | 2.6% | 5.3% | CA |  |
|  | 3.Demonstrations |  | 94.7% | 5.3% | - | CA |  |
|  | 4.Hands on activities (Assessment of Patient's Orthodontic records) |  | 100% | - | - | CA |  |
|  | 5.Clinical Clerkship/ Chairside teaching |  | 84.2% | 10.5% | 5.3% | CA |  |
|  | 6.Case presentations |  | 92.1% | 2.6% | 5.3% | CA |  |
| **Assessment Strategies** | 1.MCQs |  | 81.6% | 10.5% | 7.9% | CA |  |
|  | 2.SEQs |  | 47.4% | 18.4% | 34.2% | CNA |  |
|  | 3.Clinical Targets |  | 84.2% | 5.3% | 10.5% | CA |  |
|  | 4.OSCE |  | 94.7% | 5.3% | - | CA |  |
|  | 5.Viva Voce |  | 84.2% | 10.5% | 5.3% | CA |  |
|  | 6.TOACS |  | 89.5% | 10.5% | - | CA |  |
| **VI- Treatment of orthodontic patient- Basic concepts and goals**  **VII- Interceptive orthodontics**  **VIII- Appliances** | | | | | | | |
| **Teaching Strategies** | 1.Interactive Lecture |  | 84.2% | 7.9% | 7.9% | CA |  |
|  | 2.SGD (Tutorials, CBL) |  | 94.7% | 5.3% | - | CA |  |
|  | 3.Demonstrations |  | 84.2% | 13.2% | 2.6% | CA |  |
|  | 4.Hands on activities (Assessment of Patient's Orthodontic records) |  | 94.7% | 2.6% | 2.6% | CA |  |
|  | 5.Clinical Clerkship/ Chairside teaching |  | 92.1% | 7.9% | - | CA |  |
|  | 6.Case presentations |  | 92.1% | 7.9% | - | CA |  |
| **Assessment Strategies** | 1.MCQs |  | 81.6% | 10.5% | 7.9% | CA |  |
|  | 2.SEQs |  | 73.7% | 10.5% | 15.8% | CNA |  |
|  | 3.SAQs |  | 86.8% | 2.6% | 10.5% | CA |  |
|  | 4.OSCE |  | 94.7% | 5.3% | - | CA |  |
|  | 5.Viva Voce |  | 86.8% | 7.9% | 5.3% | CA |  |
|  | 6.DOPS |  | 86.8% | 5.3% | 7.9% | CA |  |
| **IX- Recent Advances** | | | | | | | |
| **Teaching Strategies** | 1.Interactive Lecture |  | 89.5% | 5.3% | 5.3% | CA |  |
|  | 2.Flipped classroom |  | 68.4% | 18.4% | 13.2% | CNA |  |
|  | 3.SGD |  | 81.6% | 7.9% | 10.5% | CA |  |
|  | 4.Clinical Clerkship/ Chairside teaching |  | 73.7% | 15.8% | 10.5% | CNA |  |
| **Assessment Strategies** | 1.MCQs |  | 86.8% | 5.3% | 7.9% | CA |  |
|  | 2.SAQs |  | 78.9% | 5.3% | 15.8% | CNA |  |
|  | 3.Viva Voce |  | 71.1% | 10.5% | 18.4% | CNA |  |
