# Supplementary material for: Identification of threshold concepts in the undergraduate orthodontics curriculum: a modified Delphi study
Source: BMC Med Educ. 2026 Jan 9;26:213. doi: 10.1186/s12909-025-08516-6 (PMC12882480; doi:10.1186/s12909-025-08516-6)
Supplement: Supplementary file 2 — Supplementary Material 2. [file 12909_2025_8516_MOESM2_ESM.docx]

## **Annexure I: Operational Definitions**

1. ***Transformative*:** it transforms the way a learner thinks about the discipline.
2. ***Irreversible*:** as it is unlikely that newly acquired information would be forgotten or unlearned with much difficulty after it has been accepted.
3. ***Integrative:*** understanding of the threshold concept makes new links with other topics in the subject.
4. ***Bounded:*** threshold concepts are unique to the discipline and help define its boundaries.
5. ***Troublesome:*** knowledge that is complex to understand, alien (originating from a different discourse or culture), or seemingly confused. Students may have difficulty coping with the new perspective that is offered.
6. ***Liminal*:** The Latin word "limen" means threshold. Being in a transitional state denotes being on the verge of something novel, however not quite there yet.
7. ***Reconstitutive:*** Recurrent integration and reconfiguration of knowledge occur. A student's identity or subjectivity shifts as they cross the threshold.
8. ***Discursive:*** The learner's use of language is said to elaborate once they cross the threshold.
9. ***Teaching strategies:*** A broad range of approaches that educators employ in their instruction and plan the content of their discipline.
10. ***Assessment Strategies:*** These encompasses a variety of techniques and instruments utilized by educators to evaluate the progress of their students.
11. ***Focus group Discussions (FGD):*** This is widely used in qualitative research. It is a technique in which researcher gathers participants to explore a specific topic through moderated conversation, eliciting their beliefs, attitudes and experiences.

**Annexure II: Summary of FGDs results for Identification of TCs, TS &AS.**

| **THEMES** | **THRESHOLD CONCEPTS DERIVED.** | **TEACHING STRATEGIES** | **ASSESSMENT METHODS** | **QUOTES** |
| --- | --- | --- | --- | --- |
| GROWTH & DEVELOPMENT | | | | |
|  | - Mandibular Rotations - Growth pattern of mandible and maxilla - Peak of growth indicators | SGD, Demonstrations | OSCE, Viva Voce | AR *“In growth & development the mandibular rotations, we never understood these”*  FA *“yes, it is a transformative concept”*  MS “*If we don’t tell them what is normal how will they differentiate the abnormal development*”  MS *“Growth spurt, CVM, Hand and wrist these things we cover in SGD and Demonstrations”.* |
| ORTHODONTIC ASSESSMENT AND DIAGNOSIS | | | | |
| Questionnaire/  Interview | - Chief concern of patient, - Medical/ Dental history taking, - Physical growth evaluation, | Lectures, chairside teaching, Practical  Lecture, SGDs | End of rotation test, Case presentation (Formative)  SEQs, MCQs, OSCE, Viva | AU “*Chief concern is definitely a troublesome concept; a student should know how to take medical and dental history at undergraduate level*”.  “*He should know how to identify problem, take history, evaluation of patient and after that he should know the basic treatment modality, let’s suppose Removable appliance, interception, these are the TCs*”  MS “*I think related Questions with clinical scenarios come in the paper in which its evaluation is necessary because students have to give a treatment plan along with the diagnosis*”.  MS “*We cover this in small group discussions, in demonstrations and its assessment is done via OSCE (in exam it has a station) and viva*”. |
| Clinical Evaluation | - Oral health, - Facial & dental appearance - Periodontal implications in orthodontics - Developmental disorders | Lecture, Demonstrations, clinical targets | Case presentations, Practical (OSCE) | EZ “*Yes, I think that all these are TCs based on our undergraduate curriculum*”  AR *“What is the link of ortho and Periodontology”*  MK *“Basically they teach all developmental disorders at undergraduate level but don’t tell what is their link with orthodontics”* |
| Analysis of diagnostic records | - Symmetry & space Analysis - Eyeballing method of calculating ALD - Identify COCR - Bolton Analysis, - Mixed Dentition Analysis, - Analysis of OPG - Analysis of photographic records - Link of CVM wit secondary sexual characteristics - Basic concept of CBCT | Lectures, chair side teaching, SGDs, case presentation | Clinical targets, Log book (Formative) MCQs, SEQs, OSCE (Summative) | AU *“So, in Cast Analysis, Bolton discrepancy, cast itself, arch length discrepancy in adult and mixed dentition”*  AR *“We only know that COCR exists”.*  KI *“Phrasing will be better if we write Bolton analysis instead of tooth size”*  KI *“Add OPG and Mixed dentition analysis”*  MK *“GP should know that don’t rely on CVM alone because sometimes it deceives us”.*  *AR “because it is being used a lot now a days, they should definitely know something at undergraduate level”* |
| Problem list/ Diagnosis | Formulating problem list/Diagnosis | Case presentation | Clinical targets, log Book | AU *“There is no ambiguity, problem list is therefore a Threshold concept”.*  MK *“Making an orthodontic problem list is a*  *transformative concept”* |
| **TREATMENT PLANING** | | | | |
| **BASIC CONCEPTS AND GOALS** | - Steps in planning orthodontic treatment. - Informed consent | SGDs, chair side teaching | MCQs, SEQs, OSCE, Mini-CEX | AU *“In the domains of interceptive and preventive orthodontics he will tell the steps of treatment but not in case of complex malocclusions”.*  AU “*Informed consent should be taught and priority order is also not taught this way technically it should be done by all mean”.* |
|  | - When and whom to refer |  |  | KI *“When to and whom to refer is a Threshold concept”*  AR *“In cleft lip & palate at which time patient should be referred and where”* |
|  | - Basics of biomechanics - Wire bending- 1^st^, 2^nd^, 3^rd^ order bends - Concept of anchorage - Iatrogenic effects of orthodontic treatment. | Lectures | MCQs, SEQs | RI *“It was troublesome aspect but whole biomechanics can’t be taught at undergraduate level”*  AR *“It includes 3 things, enamel demineralization, second root resorption & 3^rd^ are periodontal problems”* |
| Preventive Orthodontics | Mixed dentition problems | Lectures | MCQs, SEQs | AU *“Whole preventive and Interceptive orthodontics are a TC”*  EZ *“These all problems are threshold concepts e.g., Premature loss of deciduous teeth, retained deciduous teeth”* |
| Interceptive orthodontics | - Space management protocols- space maintenance, space regaining, space supervision, serial extractions, - Interception of Incisor trauma, - Interception of canine impaction, - Management of 1^st^ molars with poor prognosis, - Balancing & Compensating Extractions, - Etiology & early treatment of crossbite. - Etiology, diagnosis & early treatment of open bite - Molar relationship & extraction pattern | Lectures, CBLs, Demonstrations | Clinical targets, log book, MCQs, SEQs, OSCE | AU “*Before this we talked about preventive that preventive and interceptive orthodontics are threshold concepts at undergraduate level”*  AR “Finishing of a molar relationship after extraction” |
|  | Removable Appliances | Lectures, demonstrations, Practical | SEQs, SEQs, Clinical Targets, Log Book | AU “*It is our opinion that a final year/undergraduate student should be able to perform interceptive orthodontics, proper referral, take informed consent & recognize all problems to the extent that they can be sorted with removable appliances*” |
| Corrective Orthodontics | - Bracket placement - Prescription of Brackets - Class I, Class II, Class III - Clear Aligners | Lectures, chairside Teaching | MCQs, Scenario based SEQs | AR “*Definitely it is a transformative concept”*  SN “*Remove unnecessary things like universal appliances and add bracket placement”*  AU *“Class I is the only thing (in corrective orthodontics) that he (student) may attend ideally”*  AU *“It’s etiology, its features, its diagnosis, its Assessment/diagnosis, its planning and early treatment is a TC and next to it is not a TC. Referral comes after it & his(patients) consent, all these are TCs, its treatment in entirety is not a TC”*  AR *“Almost every GP is doing it …...we don’t have any idea what they are doing”* |

**Annexure III: Results of Round 1,2 and 3, Threshold Concepts.**

| **Domains & Proposed Threshold Concepts** | **Round 1**  **Percentage Agreement** | **Round 2**  **Percentage Agreement** | **McNemar p-value** | **Decision after round 2** | **Round 3**  **Percentage Agreement** | **McNemar p-value** | **Decision after round 3** |
| --- | --- | --- | --- | --- | --- | --- | --- |
| **I Patient’s information** | | | | | | | |
| 1.Taking history of Orthodontic patient | 95.1% | 94.7% | 1 | CA-Not sent to Round 3 |  |  | **CA-It is a TC** |
| **II Patient’s Clinical Evaluation** | | | | | | | |
| 2.Soft tissue paradigm | 82.9% | 92.1% | .344 | CA-Not sent to Round 3 |  |  | **CA-It is a TC** |
| 3. Facial profile analysis | 87.8% | 86.8% | 1 | CA-Not sent to Round 3 |  |  | **CA-It is a TC** |
| **III Growth and development** | | | | | | | |
| 4.Growth pattern of Mandible and Maxilla | 97.6% | 100% | Cannot be computed | CA-Not sent to Round 3 |  |  | **CA-It is a TC** |
| 5.Significance of mandibular rotations | 82.9% | 86.8% | 1 | CA-Not sent to Round 3 |  |  | **CA-It is a TC** |
| 6.Various growth indicators determining the peak skeletal growth/Growth Assessment parameters | 92.7% | 94.7% | 1 | CA-Not sent to Round 3 |  |  | **CA-It is a TC** |
| **IV Analysis of diagnostic records** | | | | | | | |
| 7.Arch dimensions (Arch length, width, depth & Curve of Wilson, Spee, Monson | 82.9% | 89.5% | .549 | CA-Not sent to Round 3 |  |  | **CA-It is a TC** |
| 8.Eyeballing method of calculating ALD | 75.6% | 68.4% | .629 | CNA-sent to Round 3 (Rephrased) | 74.3% | **.**754 | **CNA-It is not a TC** |
| 9.Bolton’s analysis | 78% | 84.2% | .581 | CA-Not sent to Round 3 |  |  | **CA-It is a TC** |
| 10.OPG analysis/Dental age analysis of OPG | 90.2% | 97.4% | .375 | CA-Not sent to Round 3 |  |  | **CA-It is a TC** |
| 11.Analysis of CVM stages on lateral Cephalogram | 87.8% | 94.7% | .687 | CA-Not sent to Round 3 |  |  | **CA-It is a TC** |
| 12.Limitations of ANB angle | 56.1% | 71.1% | .210 | CNA-sent to Round 3 | 71.4% | 1 | **CNA-It is not a TC** |
| 13.Composite Mixed dentition analysis | 75.6%  Rephrased | 92.1% | .065 | CA-Not sent to Round 3 |  |  | **CA-It is a TC** |
| **V Diagnosis** | | | | | | | |
| 14.Making a problem list | 87.8% | 89.5% | 1 | CA-Not sent to Round 3 |  |  | **CA-It is a TC** |
| 15.Diagnosis of growth and developmental disorders | 85.4% | 84.2% | 1 | CA-Not sent to Round 3 |  |  | **CA-It is a TC** |
| 16.Diagnosis of impacted maxillary canines. | 87.8% | 89.5% | 1 | CA-Not sent to Round 3 |  |  | **CA-It is a TC** |
| **VI Treatment of orthodontic patient- Basic concepts and goals** | | | | | | | |
| 17.Treatment planning of various Malocclusions | 85.4% | 92.1% | .508 | CA-Not sent to Round 3 |  |  | **CA-It is a TC** |
| 18.Significance of molar (healthy/mutilated) relationship | 95.1% | 100% | Cannot be computed | CA-Not sent to Round 3 |  |  | **CA-It is a TC** |
| 19.Extraction patterns (molar/premolar/Incisor) in treatment planning | 87.8% | 84.2% | .727 | CA-Not sent to Round 3 |  |  | **CA-It is a TC** |
| 20. Balancing and compensating extractions of 6s. | 70.7% | 86.8% | .118 | CA-Not sent to Round 3 |  |  | **CA-It is a TC** |
| 21.Periodontal considerations for orthodontic treatment | 85.4% | 86.8% | 1 | CA-Not sent to Round 3 |  |  | **CA-It is a TC** |
| 22.Basics of biomechanics | 90.2% | 89.5% | 1 | CA-Not sent to Round 3 |  |  | **CA-It is a TC** |
| 23.Basics of Anchorage control in orthodontics | 95.1% | 94.7% | 1 | CA-Not sent to Round 3 |  |  | **CA-It is a TC** |
| 24.Basics of Retention and Relapse | 97.6% | 100% | Cannot be computed | CA-Not sent to Round 3 |  |  | **CA-It is a TC** |
| 25.Clinical Implications of developmental disorders in orthodontics | 82.9% | 81.6% | 1 | CA-Not sent to Round 3 |  |  | **CA-It is a TC** |
| 26.Knowing when and whom to refer | 95.1% | 97.4% | 1 | CA-Not sent to Round 3 |  |  | **CA-It is a TC** |
| 27.Iatrogenic effects of orthodontic treatment | 90.2% | 92.1% | 1 | CA-Not sent to Round 3 |  |  | **CA-It is a TC** |
| **VII Interceptive orthodontics** | | | | | | | |
| 28.Management of 1st molars with poor prognosis | 80.5% | 89.5% | .388 | CA-Not sent to Round 3 |  |  | **CA-It is a TC** |
| 29.Interception of incisor Trauma | 90.2% | 89.5% | 1 | CA-Not sent to Round 3 |  |  | **CA-It is a TC** |
| 30.Mixed dentition space management protocols (space maintenance/regaining, supervision/serial extraction) | 82.9% | 92.1% | .344 | CA-Not sent to Round 3 |  |  | **CA-It is a TC** |
| **VIII Appliances** | | | | | | | |
| 31.Indications of various appliances (Removable/functional/fixed) | 90.2% | 86.8% | 1 | CA-Not sent to Round 3 |  |  | **CA-It is a TC** |
| 32.Bracket placement | 68.3% | 65.8% | 1 | CNA-sent to Round 3 | 74.3% | .815 | **CNA-It is not a TC** |
| 33.Prescriptions of brackets | 36.6% | 34.2% | 1 | CNA-sent to Round 3 | 51.4% | .238 | **CNA-It is not a TC** |
| **IX Recent Advances** | | | | | | | |
| 34.Concept of clear aligners | 65.9% | 71.1% | 1 | CNA-sent to Round 3 (Rephrased) | 71.4% | 1 | **CNA-It is not a TC** |
| 35.Basic concept of CBCT | 80.5% | 86.8% | .549 | CA-Not sent to Round 3 |  |  | **CA-It is a TC** |
| **X Concepts Suggested by Panelists in Round 1** | | | | | | | |
| 1. Occlusion |  | 97.4% |  |  | 97.1% | 1 | **CA-It is a TC** |
| 2.Etiology of Malocclusion |  | 92.1% |  |  | 91.4% | 1 | **CA-It is a TC** |
| 3.Biology of tooth movement |  | 92.1% |  |  | 91.4% | 1 | **CA-It is a TC** |
| 4.Ethical practice |  | 92.1% |  |  | 94.3% | 1 | **CA-It is a TC** |

| **CA Consensus Achieved**  **CNA Consensus not Achieved**  **TC Threshold Concept** |
| --- |

**Annexure IV: Results of Round 1,2 and 3, Teaching & Assessment Strategies under 9 domains for TCs presented in table above**

| **Domains & Proposed Strategies from Round 1** | | **Round 2 Percentage Agreement** | **Round 3 Percentage Agreement** | **McNemar p-value** | **Decision after round 3** |
| --- | --- | --- | --- | --- | --- |
| **I-Patient’s information**  **II- Patient’s Clinical Evaluation** | | | | | |
| Teaching Strategies | 1.Clinical clerkship/Chair side teaching | 97.4% | 97.1% | 1 | CA-Recommended TS |
|  | 2.SGD (simulated patients, Role play, tutorial, CBL) | 97.4% | 97.1% | 1 | CA-Recommended TS |
|  | 3. Demonstration | 92.1% | 91.4% | 1 | CA-Recommended TS |
|  | 4. Case presentation | 92.1% | 91.4% | 1 | CA-Recommended TS |
|  | 5.Interactive lecture | 78.9% | 94.3% | .109 | CA-Recommended TS |
| **Assessment Strategies** | 1.MCQs | 73.7% | 77.1% | 1 | CNA-Excluded from the list |
|  | 2.SEQs | 44.7% | 65.7% | .077 | CNA-Excluded from the list |
|  | 3.Viva Voce | 78.9% | 80% | 1 | CA-Recommended AS |
|  | 4.Mini-CEX | 76.3% | 88.6% | .508 | CA-Recommended AS |
|  | 5.OSCE | 94.7% | 94.3% | 1 | CA-Recommended AS |
|  | 6.TOACS | 92.1% | 94.3% | 1 | CA-Recommended AS |
| **III- Growth and development** | | | | | |
| **Teaching Strategies** | 1.Interactive Lecture | 97.4% | 97.1% | 1 | CA-Recommended TS |
|  | 2.SGD (Tutorials, CBL) | 92.1% | 91.4% | 1 | CA-Recommended TS |
|  | 3.Demonstrations | 63.2% | 71.4% | .481 | CNA-Excluded from the list |
|  | 4.Clinical Clerkship (Assessment of Patient's Orthodontic records) | 76.3% | 74.3% | 1 | CNA-Excluded from the list |
|  | 5.Flipped Classroom | 73.7% | 74.3% | 1 | CNA-Excluded from the list |
| **Assessment Strategies** | 1.MCQs | 92.1% | 94.3% | 1 | CA-Recommended AS |
|  | 2.SAQs | 81.6% | 80% | 1 | CA-Recommended AS |
|  | 3.SEQs | 65.8% | 77.1% | .227 | CNA-Excluded from the list |
|  | 4.OSCE | 81.6% | 85.7% | 1 | CA-Recommended AS |
|  | 5.Viva Voce | 86.8% | 85.7% | 1 | CA-Recommended AS |
| **IV-** **Analysis of diagnostic records**  **V- Diagnosis** | | | | | |
| **Teaching Strategies** | 1.Interactive Lecture | 71.1% | 88.6% | .065 | CA-Recommended TS |
|  | 2.SGD (Tutorials, CBL) | 92.1% | 91.4% | 1 | CA-Recommended TS |
|  | 3.Demonstrations | 94.7% | 97.1% | 1 | CA-Recommended TS |
|  | 4.Hands on activities (Assessment of Patient's Orthodontic records) | 100% | 100% | Cannot be computed | CA-Recommended TS |
|  | 5.Clinical Clerkship/ Chairside teaching | 84.2% | 88.6% | 1 | CA-Recommended TS |
|  | 6.Case presentations | 92.1% | 94.3% | 1 | CA-Recommended TS |
| **Assessment Strategies** | 1.MCQs | 81.6% | 85.7% | 1 | CA-Recommended AS |
|  | 2.SEQs | 47.4% | 68.6% | .115 | CNA-Excluded from the list |
|  | 3.Clinical Targets | 84.2% | 88.6% | 1 | CA-Recommended AS |
|  | 4.OSCE | 94.7% | 97.1% | 1 | CA-Recommended AS |
|  | 5.Viva Voce | 84.2% | 82.9% | 1 | CA-Recommended AS |
|  | 6.TOACS | 89.5% | 91.4% | 1 | CA-Recommended AS |
| **VI-** **Treatment of orthodontic patient- Basic concepts and goals**  **VII- Interceptive orthodontics**  **VIII- Appliances** | | | | | |
| **Teaching Strategies** | 1.Interactive Lecture | 84.2% | 82.9% | 1 | CA-Recommended TS |
|  | 2.SGD (Tutorials, CBL) | 94.7% | 94.3% | 1 | CA-Recommended TS |
|  | 3.Demonstrations | 84.2% | 82.9% | 1 | CA-Recommended TS |
|  | 4.Hands on activities (Assessment of Patient's Orthodontic records) | 94.7% | 94.3% | 1 | CA-Recommended TS |
|  | 5.Clinical Clerkship/ Chairside teaching | 92.1% | 91.4% | 1 | CA-Recommended TS |
|  | 6.Case presentations | 92.1% | 91.4% | 1 | CA-Recommended TS |
| **Assessment Strategies** | 1.MCQs | 81.6% | 82.9% | 1 | CA-Recommended AS |
|  | 2.SEQs | 73.7% | 74.3% | 1 | CNA-Excluded from the list |
|  | 3.SAQs | 86.8% | 85.7% | 1 | CA-Recommended AS |
|  | 4.OSCE | 94.7% | 94.3% | 1 | CA-Recommended AS |
|  | 5.Viva Voce | 86.8% | 85.7% | 1 | CA-Recommended AS |
|  | 6.DOPS | 86.8% | 88.6% | 1 | CA-Recommended AS |
| **IX- Recent Advances** | | | | | |
| **Teaching Strategies** | 1.Interactive Lecture | 89.5% | 88.6% | 1 | CA-Recommended TS |
|  | 2.Flipped classroom | 68.4% | 68.6% | 1 | CNA-Excluded from the list |
|  | 3.SGD | 81.6% | 82.9% | 1 | CA-Recommended TS |
|  | 4.Clinical Clerkship/ Chairside teaching | 73.7% | 82.9% | .344 | CA-Recommended TS |
| **Assessment Strategies** | 1.MCQs | 86.8% | 88.6% | 1 | CA-Recommended AS |
|  | 2.SAQs | 78.9% | 65.7% | .359 | CNA-Excluded from the list |
|  | 3.Viva Voce | 71.1% | 74.3% | 1 | CNA-Excluded from the list |

| **CA Consensus Achieved**  **CNA Consensus not Achieved**  **TS Teaching Strategy**  **AS Assessment Strategy** |
| --- |
