# Supplementary material for: Identification of threshold concepts in the undergraduate orthodontics curriculum: a modified Delphi study
Source: BMC Med Educ. 2026 Jan 9;26:213. doi: 10.1186/s12909-025-08516-6 (PMC12882480; doi:10.1186/s12909-025-08516-6)
Supplement: Supplementary file 3 — Supplementary Material 3. [file 12909_2025_8516_MOESM3_ESM.docx]

**I-Patient’s information**

**II- Patient’s Clinical Evaluation**


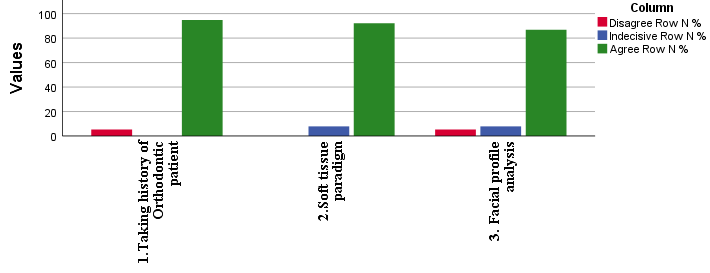


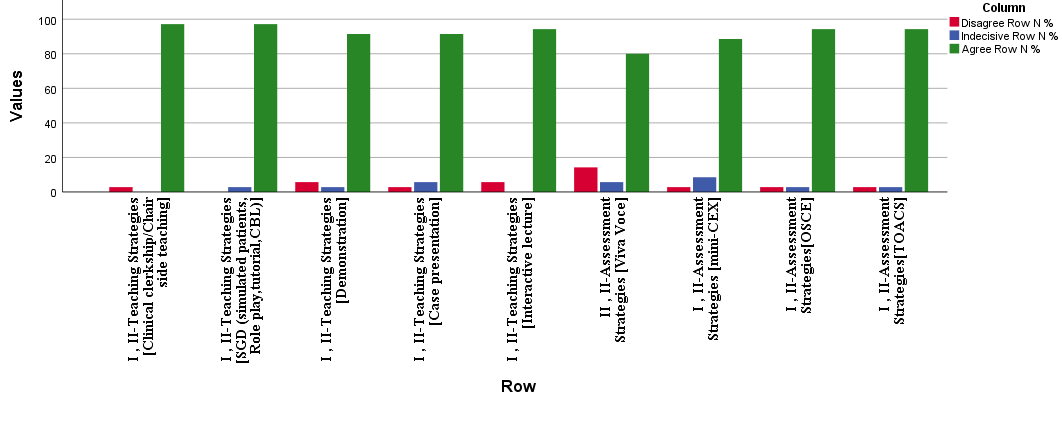


**Figure 5.1: Accepted items as TCs, TS and AS, domains I and II.**


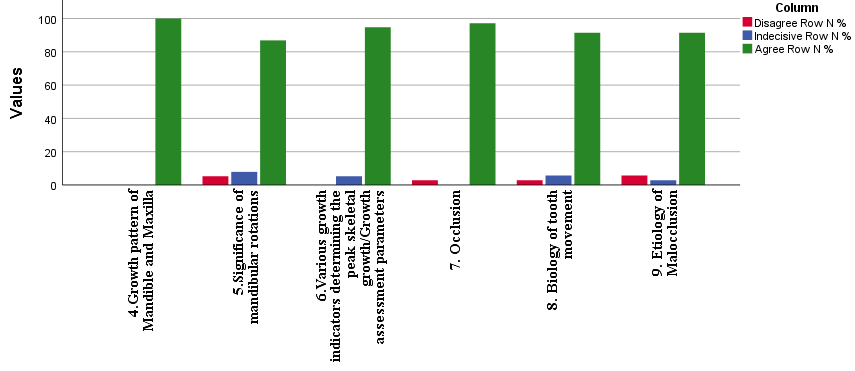


**III- Growth and development**


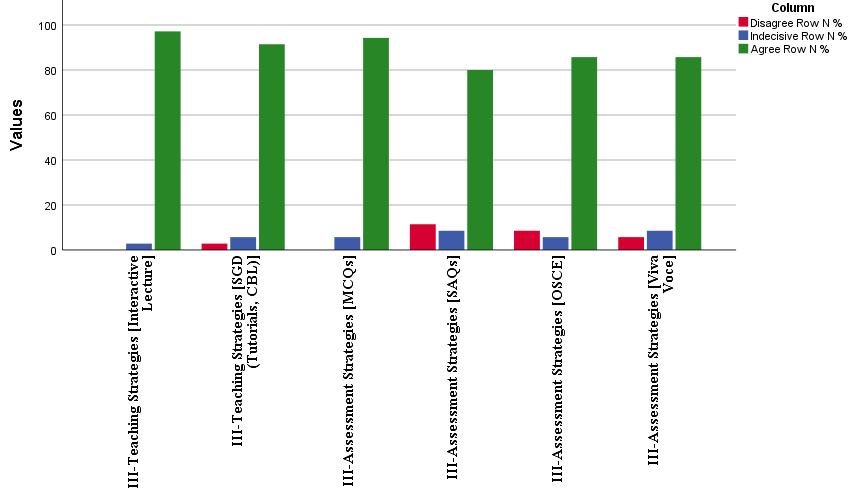


**Figure 5.2: Accepted items as TCs, TS and AS, domain III.**

**IV- Analysis of diagnostic records**

**V- Diagnosis**


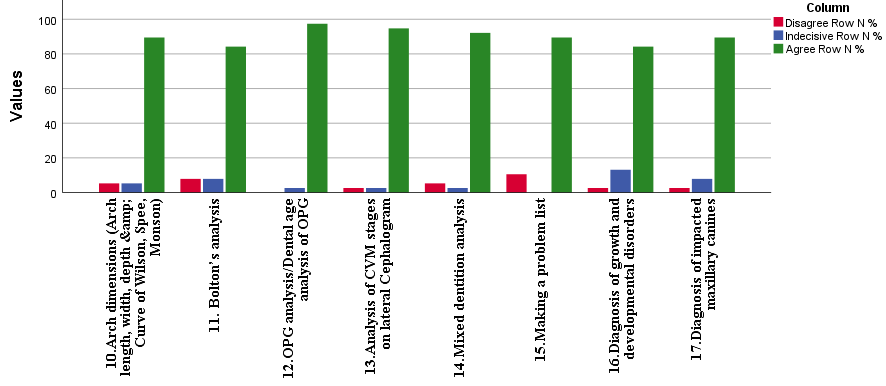

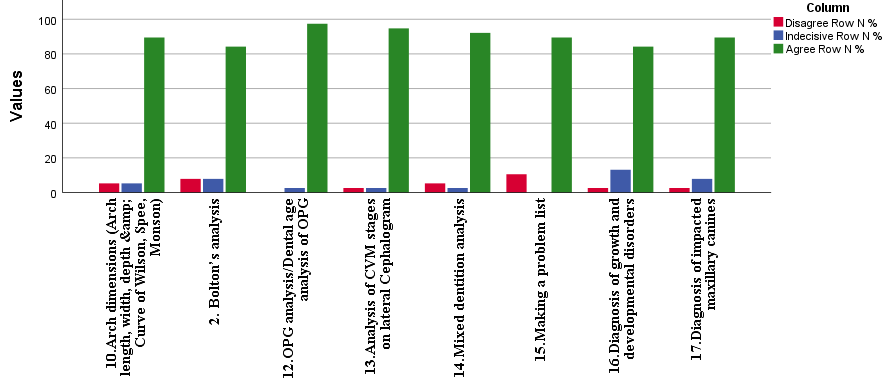


**Figure 5.3: Accepted items as TCs, TS and AS, domains IV and V.**


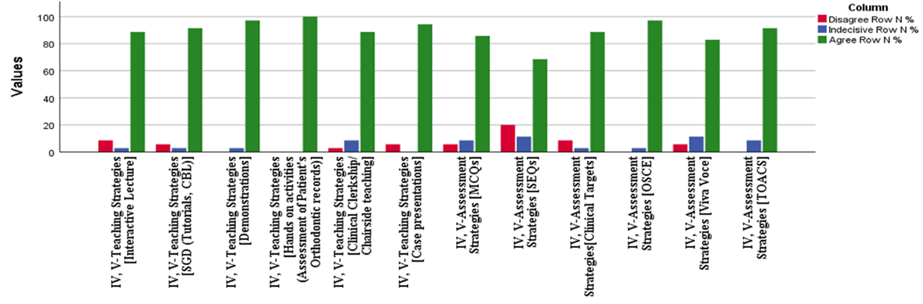

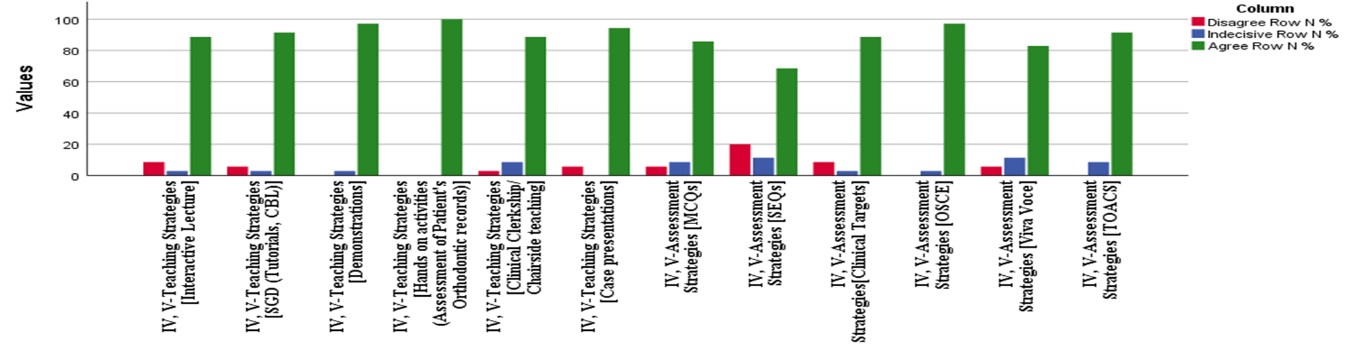


**VI- Treatment of orthodontic patient- Basic concepts and goals**

**VII- Interceptive orthodontics**

**VIII- Appliances**


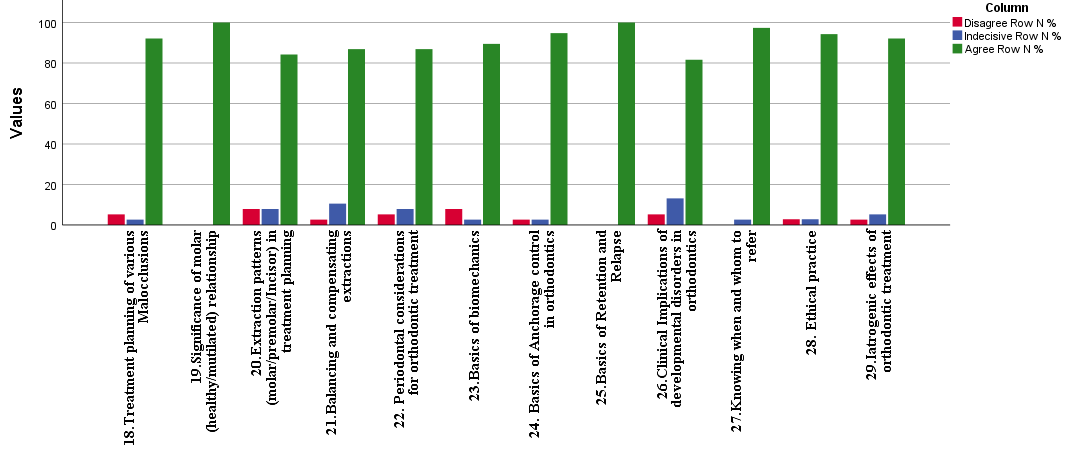


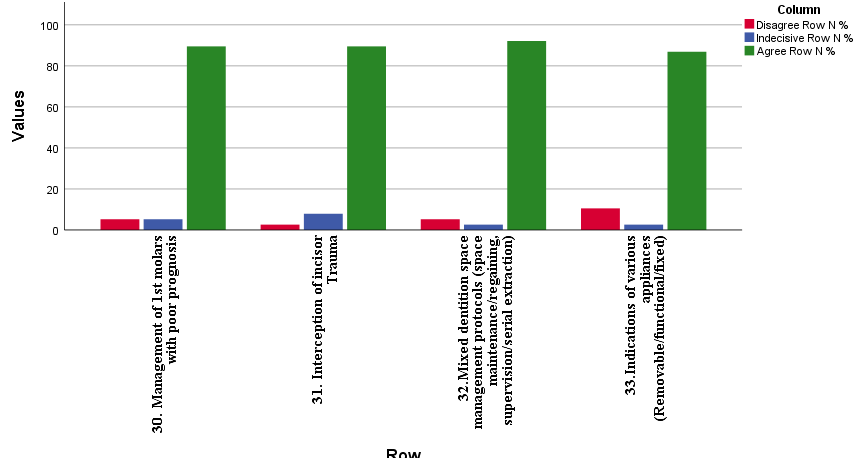


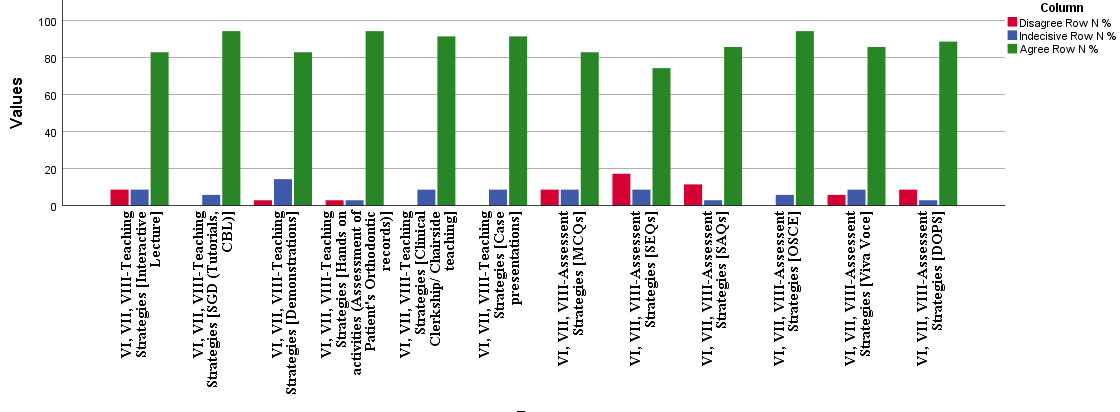


**Figure 5.4: Accepted items as TCs, TS and AS, domains VI, VII and VIII.**

**IX- Recent Advances**


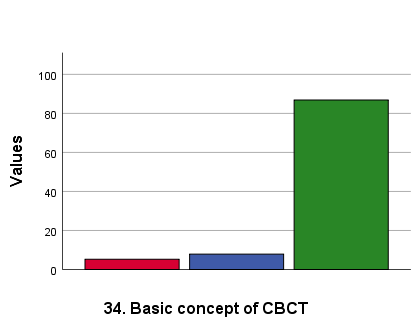


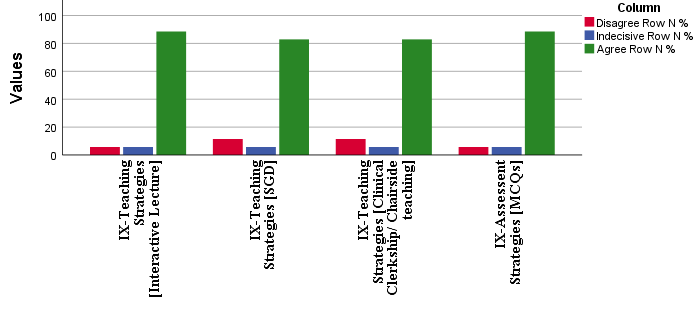


**Figure 5.5: Accepted items as TCs, TS and AS, domain IX.**
